# Interpreting *Prevotella* and *Bacteroides* as biomarkers of diet and lifestyle

**DOI:** 10.1186/s40168-016-0160-7

**Published:** 2016-04-12

**Authors:** Anastassia Gorvitovskaia, Susan P. Holmes, Susan M. Huse

**Affiliations:** Brown University, Providence, RI USA; Sequoia Hall, Stanford University, Stanford, CA USA; Indigo Agriculture Inc, Cambridge, MA USA

**Keywords:** Microbiome, Biomarkers, Enterotypes, Human gut

## Abstract

**Background:**

In a series of studies of the gut microbiome, “enterotypes” have been used to classify gut microbiome samples that cluster together in ordination analyses. Initially, three distinct enterotypes were described, although later studies reduced this to two clusters, one dominated by *Bacteroides* or Clostridiales species found more commonly in Western (American and Western European) subjects and the other dominated by *Prevotella* more often associated with non-Western subjects. The two taxa, *Bacteroides* and *Prevotella*, have been presumed to represent consistent underlying microbial communities, but no one has demonstrated the presence of additional microbial taxa across studies that can define these communities.

**Results:**

We analyzed the combined microbiome data from five previous studies with samples across five continents. We clearly demonstrate that there are no consistent bacterial taxa associated with either *Bacteroides*- or *Prevotella*-dominated communities across the studies. By increasing the number and diversity of samples, we found gradients of both *Bacteroides* and *Prevotella* and a lack of the distinct clusters in the principal coordinate plots originally proposed in the “enterotypes” hypothesis. The apparent segregation of the samples seen in many ordination plots is due to the differences in the samples’ *Prevotella* and *Bacteroides* abundances and does not represent consistent microbial communities within the “enterotypes” and is not associated with other taxa across studies. The projections we see are consistent with a continuum of values created from a simple mixture of *Bacteroides* and *Prevotella*; these two biomarkers are significantly correlated to the projection axes. We suggest that previous findings citing *Bacteroides*- and *Prevotella*-dominated clusters are the result of an artifact caused by the greater relative abundance of these two taxa over other taxa in the human gut and the sparsity of *Prevotella* abundant samples.

**Conclusions:**

We believe that the term “enterotypes” is misleading because it implies both an underlying consistency of community taxa and a clear separation of sets of human gut samples, neither of which is supported by the broader data. We propose the use of “biomarker” as a more accurate description of these and other taxa that correlate with diet, lifestyle, and disease state.

**Electronic supplementary material:**

The online version of this article (doi:10.1186/s40168-016-0160-7) contains supplementary material, which is available to authorized users.

## Background

The gut microbiome plays an important role in human health and disease. Understanding what constitutes a health-promoting (eubiotic) or disease-promoting (dysbiotic) microbial community has become the focus of significant research. In 2011, Arumugam et al. [1] postulated that all human gut microbiomes could be classified as one of three distinct bacterial communities or “enterotypes”^1^. The researchers used ordination analysis on three datasets: a set of 33 European, Japanese, and American subjects, a set of 85 Danish, and a set of 154 Americans. Each dataset independently displayed a similar clustering pattern with subjects dominated by one of three different taxa: Bacteroides, Provetella, or members of the order Clostridiales. The authors speculated that if this clear separation of clusters were evident across all human gut samples, people could be classified by their enterotype, which could in turn be used to guide diagnostics and treatment options. Only one of datasets showed clear separation between the *Bacteroides*- and Clostridiales-dominated samples, while two showed overlapping points consistent with a gradient.

Since the introduction of the enterotypes hypothesis, several studies have evaluated their presence in other human gut communities. The first such study by Wu et al. [2] sequenced 98 Americans and used multiple clustering techniques but found only two of the three original clusters: *Bacteroides* and *Prevotella*, laying at either end of a *Prevotella*-*Bacteroides* gradient. The third cluster was not distinct in their data, with *Ruminococcus* (a Clostridiales genus) equally abundant in both of the first two enterotypes. They compared their findings with a previously published study on Italian and rural African children wherein Italian children had *Bacteroides*-dominated gut microbiomes and African children *Prevotella*-dominated gut microbiomes. As part of our own analysis of over 200 healthy American subjects enrolled in the Human Microbiome Project (HMP) [3], we confirmed the results of Wu et al. that two of the three clusters were not distinct. Samples high in *Bacteroides* or members of the order Clostridiales, including *Ruminococcus*, form a continuum, rather than discrete clusters. While the *Prevotella*-dominated samples did cluster separately with the HMP data, we speculated that as more samples and studies were evaluated, the separation between clusters would diminish, and gut communities would appear as a continuum of abundance gradients between *Bacteroides*, *Prevotella*, and various members of the order Clostridiales. In a review article published soon after, Jeffrey et al. [4] reached the same conclusion that a gradient of microbial populations was a more accurate interpretation of the data.

Several studies thereafter sought to re-examine enterotypes using more careful clustering analyses [5–7]. Overall, these additional studies demonstrated that with increased statistical rigor, support remained for only two community types: one dominated by *Prevotella* and one dominated by *Bacteroides* or members of the Firmicutes [5]. Additionally, the utility of enterotypes to classify patients was shown to be limited given that individuals can shift between *Bacteroides*/Firmicutes- and *Prevotella*-dominated communities over time, with as many as 30 % of samples in one dataset shifting between enterotypes [6, 7]. Knights et al. [6] further cautioned that supervised ordination methods can lead to false clustering when the number of features in the data (here microbial genera) is much higher than the number of samples, as is the case in most human gut microbiome studies. Overall, these studies found that the weight of evidence did not support the classification of human gut microbiomes into discrete and stable enterotypes.

Despite the ongoing refutation of the appropriateness and stability of enterotypes, researchers continue to use them to interpret ordination analyses that separate *Prevotella*-dominated communities from those dominated by *Bacteroides*, Clostridiales, and a fourth taxon, *Bifidobacterium*. These communities recur across various study populations and diets. For instance, in a study that predated the enterotypes hypothesis, De Filippo et al. [8] studied 29 children, 15 from Italy and 14 from Burkino Faso. The gut microbiomes of the Burkino Faso children were mostly dominated by *Prevotella*, while the Italian children were mostly dominated by *Bacteroides* and Clostridiales. Both populations included a few subjects dominated by Actinobacteria, mainly *Bifidobacterium*, a beneficial microbe associated with milk-based diets such as breast-feeding [9]. The Burkino Faso diet was rich in vegetables and fiber, and children were breast-fed up to 2 years of age. The diet of the Italian children was a “Western diet” low in fiber, incorporating more animal protein, fat, and sugar, and children tended to be breast-fed for only 1 year. It was postulated that diet could be a driving factor in shaping the gut microbiome.

Ou et al. [10] compared twelve African Americans and twelve native Africans as part of a study examining the higher prevalence of colon cancer in Americans. The populations clustered distinctly, with the African Americans dominated by *Bacteroides* and the native Africans by *Prevotella*. The native African diet was high in complex fiber, while the African American diet was a Western diet high in protein. Yatsunenko et al. [11] compared gut microbiome samples of healthy adults and children from Malawi, Venezuela, and the USA. They found a gradient of *Bacteroides* and *Prevotella* in both adults and children over 6 months. They also found a discrete group of samples dominated by *Bifidobacterium*, in children under 6 months of age, which is consistent with its association with breast-feeding. The US subjects clustered separately from the Malawian and Venezuelans, who had relatively more *Prevotella*.

In a comparison of 50 metropolitan and 46 rural Russians, Tyakht et al. [12, 13] found between two and three community types. *Prevotella* accounted for one of the clusters and was prevalent in samples from one of the rural villages, but Firmicutes (rather than the combination of *Bacteroides* and Firmicutes) and *Bifidobacterium* dominated the remaining samples. The ingredients of the diet eaten by the metropolitan subjects were similar to those in a Western diet, but the sources of their food were very different. The authors speculated that the lack of *Bacteroides*-dominated samples could be due to the metropolitan Russian food sources, which were homegrown rather than industrially processed. Overall, the results from these various studies are consistent. The gut microbiomes of Americans and Europeans having a Western diet tend to be dominated by *Bacteroides* and Clostridiales, while rural populations with a high fiber, low-protein diet tend to be dominated by *Prevotella*.

Despite the frequent use of the term “enterotype” to describe clustering of the gut microbiomes of research subjects from different cultures and with different dietary habits, the membership of the microbial communities associated with the enterotypes has not been clearly defined. *Prevotella*, *Bacteroides*, Clostridiales, and *Bifidobacterium* are recurring taxa, but a deeper understanding of the community structure of samples dominated by these taxa across continents and cultures has not been undertaken. Whether distinct community types or gradients, stable over time or not, we do not have a functional description of what other bacteria comprise the *Prevotella*- or *Bacteroides*/Clostridiales-dominated clusters beyond the dominant taxon. Here we present an analysis of the combined data from these published studies to characterize the broader community membership of the two primary enterotypes, so that we might better understand what these communities can tell us about the human gut microbiome.

## Results and discussion

Our analysis compiled 747 samples from five studies encompassing 484 genera. We included 126 samples from the Arumugam et al. [14]: 41 mixed Europe and Asia, 85 Europeans, 24 Native African and African American samples [10], 96 urban and rural Russians samples [12], 210 samples of healthy American adults from the Human Microbiome Project [15], and 291 adult samples from Malawi, Venezuela, and America [11].

### Multivariate analysis

**Fig. 1:**
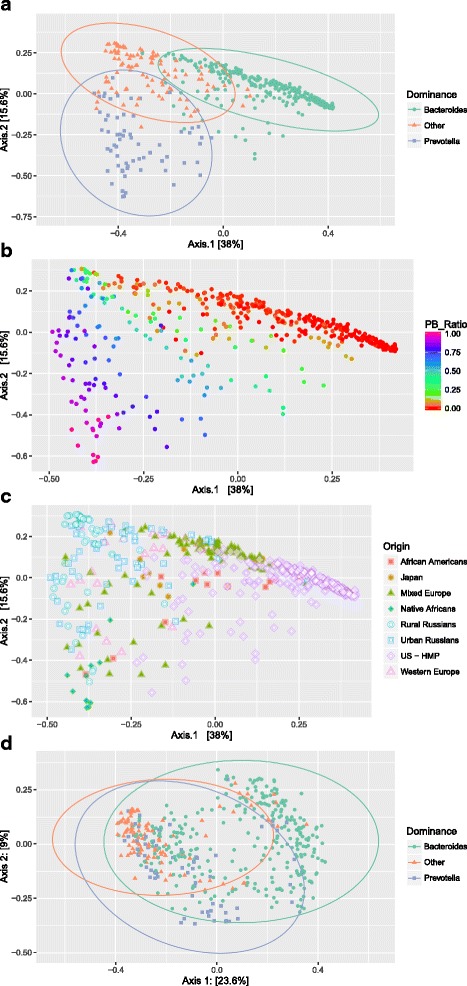
PCoA plots using the Bray distance metric with all the samples except for the Yatsunenko study. **a** Samples colored by their most prominent taxon. If the sample is dominated neither by *Prevotella* nor *Bacteroides*, it is classified as other. Ellipses were projected for each group in the plot. The *ellipse axes* represent the directions of the within-group covariance matrices, and their bounds represent two standard deviations in each direction from the cluster mean. **b** Samples are colored by their value for the Prevotella ratio (relative abundance of *Prevotella*/[*Bacteroides* + *Prevotella*]) on a spectrum with red indicating no *Prevotella* and purple no *Bacteroides*. **c** Samples are colored by population of origin. **d** The Bray distance has been recalculated without the relative abundances of *Bacteroides* and *Prevotella*. Samples are colored by most prominent taxon in the original samples distributions, and ellipses were projected for each group in the plot (same as in plot **a**)

The “enterotype” hypothesis was initially proposed based on clustering results seen in a principal coordinates analysis. In our initial ordination analysis, we found that all three populations from the Yatsunenko study clustered separately from the remaining studies (Additional file 1: Figure S1). We therefore performed our primary multivariate analyses without these samples but then confirmed our findings with an independent analysis of them. Combined samples from the four remaining datasets do not cluster discretely with either metric or non-metric multidimensional scaling (PCoA^2^ or NMDS^3^), using Bray-Curtis or Morisita-Horn distances, but instead show a gradient across both dominant taxa and study populations (Fig. 1, Additional file 2: Figure S10). Coloring by dominant taxa (Fig. 1a) and the *Prevotella* ratio (the ratio of *Prevotella* to the sum of *Prevotella* and *Bacteroides*) (Fig. 1b) illustrate the relationship of axes 1 and 2 to *Bacteroides* (toward the upper right) and *Prevotella* (toward the lower left) in the samples. When colored by population, we see that the second axis separates the samples within a population, as does the first axis but to a lesser extent (Fig. 1c). In PCoA analysis of the Malawi, Venezuela, and US populations from the Yatsunenko study (Additional file 3: Figure S7), the first axis appears to correspond primarily to the *Prevotella* ratio. The three countries are spread along the second axis with the US samples separating from the other two, but the Malawian and Venezuelan samples are intermingled. In general, these data do segregate along the first axis, with fewer intermediate samples than in the combined studies, but distinct clustering is unclear. These ordination results show that the primary axes continue to correlate with *Bacteroides* and *Prevotella* when the studies are combined.

To assess the persistent clustering of the microbial communities independent of the relative abundances of *Bacteroides* and *Prevotella*, we removed these two taxa from all the samples and reran the PCoA analysis (Fig. 1d, Additional file 3: Figure S7B). Without *Prevotella* and *Bacteroides*, the original microbial community classifications (“enterotypes”) no longer segregate the samples. The ellipses in Figs. 1a and 1d were constructed as confidence regions for each of the groups; these regions would represent 95 % of the points if the bivariate data were Gaussian. This demonstrates the effect on the clustering of removing the two dominant taxa. When the dominant taxa are included, the ellipses abut without overlapping. When the dominant taxa are removed, two of the ellipses are almost completely within the third, and all but three of the *Prevotella*-dominated samples are contained within the ellipse delineating the central *Bacteroides* samples. Separation of the original clusters is no longer present, and the remaining taxa now account for only 33 % of the variability along axes 1 and 2, as opposed to 54 %. The ordinations of the full samples appear to be driven primarily by the relative quantities of these two highly abundant taxa and not by the presence of distinct microbial communities associated with each group. Interestingly, there is still clustering structure and differentiation of samples visible in Fig. 1d, as one would expect when comparing samples from patients in different geographic regions having different lifestyles and diets. These data structures, however, no longer correlate clearly with *Bacteroides* and *Prevotella*.

### Taxonomic components of microbial communities

To identify taxa integral to a broader definition of microbial community structures, we looked for taxa that correlated with the dominant genus (*Prevotella* or *Bacteroides*) across studies (Additional file 4: Table S1). Several researchers reported correlations within *Bacteroides*-dominated samples within their studies, including *Acidminococcus*, *Roseburia*, *Faecalibacterium*, *Anaerostipes*, *Parabacteroides*, and *Clostridiales* [14], *Alistipes* only [16], *Escherichia* (*Enterobacteriaceae*) and *Acinetobacter* [10], and *Faecalibacterium* and *Enterobacteriaceae* [8]. None of these associations, however, were apparent across all studies. In fact, only *Faecalibacterium* and *Enterobacteriaceae* appear in even two of the four studies that reported correlation analyses. No correlations were found within the *Prevotella*-dominated samples in more than one previous study. Prevotella was associated with *Streptococcus*, *Enterococcus*, *Desulfovibrio*, and *Lachnospiraceae* [14], *Succinivibrio* and *Oscillospira* [10], and *Xylanibacter* and *Butyrivibrio* [8]. The majority of taxa found in the Russian communities [12, 13] were not found in previously reported studies. Tyakht et al. used triplets of the three most abundant members of their various samples to compare with these other studies and found that 43 % of their samples were dominated by triplets not found in the non-Russian groups.

To confirm this finding of no consistent multi-study taxa associated with either dominant taxon, and to increase our statistical power of detection, we combined the samples from all of the studies and reanalyzed them. We used the Spearman coefficient (a nonparametric method used in most of the previous studies) as well as SparCC [17]. Using Spearman on all the data and the DESeq2 [18] negative binomial test on the HMP data, we found no taxa in the combined set of *Prevotella*-dominated samples that correlated with *Prevotella* above our threshold for statistical and biological significance (Additional file 4: Table S1). SparCC did find two Clostridiales genera that correlated with *Prevotella* in the combined dataset, *Ruminococcus* (*R*^2^ = 0.77) and *Dialister* (*R*^2^ = 0.73), but neither of these had a significant correlation in any single study. In the combined *Bacteroides*-dominated samples, we did not find any significant correlations using the Spearman correlation. A few Clostridiales taxa and one Bacteroidales genus in individual studies did seem correlated with *Bacteroides*, but none of these were found in more than two of the studies. Using SparCC, *Subdoligranulum* (*R*^2^ = 0.75) and *Faecalibacterium* (*R*^2^ = 0.73) correlated with *Bacteroides* but only in the combined set, not in any of the individual datasets. No taxa were significant in more than one of the studies when analyzed separately. These data do highlight that members of both Bacteroidales and Clostridiales are prominent members of the human gut microbiome of Americans and Western Europeans. To explore additional taxonomic differences between groups defined in the Yatsunenko and HMP studies as *Prevotella*-rich and *Bacteroides*-rich, we used the package DESeq2 and the tests based on variance transformed data using the negative binomial model (DESeq function with default arguments) as explained in [18]. This provided a ranking of the most differentially abundant taxa. The analysis of the Yatsunenko data showed *Parabacteroides*, *Alistipes*, and *Subdoligranulum*, to be more prevalent in *Bacteroides*-dominated samples, but these results were not reflected in the HMP data. The DESeq2 analysis confirmed the previous analyses, showing no taxa consistently associating with the *Bacteroides*- and *Prevotella*-dominated samples. Recent work by Lovell et al. [19] has emphasized the importance of proportionality analysis when working with relative abundance data. Applying their proposed algorithm on our compiled data, we still found no significant proportionalities across studies. The taxa that had proportionality statistics less than 0.01 were either very rare or even possibly artifacts of the sequencing process. Two of these taxa were found in only one sample, and all of the others were identified in only populations from one study (the European samples from the Arumugam study). They are not prevalent in either the *Prevotella*- or *Bacteroides*-dominated samples, and in fact, are absent from the majority of both. These taxa are clearly not important functional components or biomarkers of a human gut community. The analysis itself can be found in the Additional file 5 containing the Rmd commands, data and output. This file contains all the code the reader could require to follow the same workflow on a different data set.

**Fig. 2:**
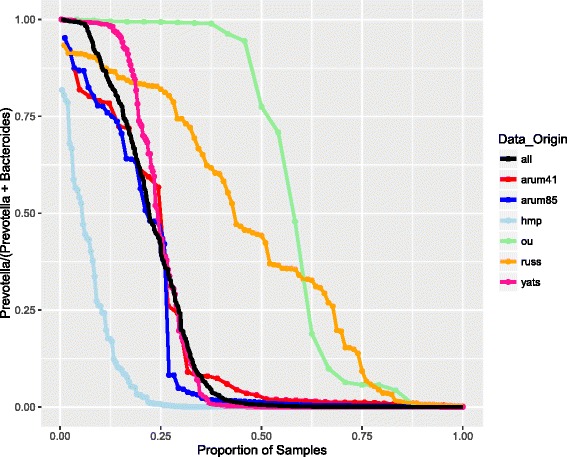
Prevotella ratio distributions (relative abundance of *Prevotella*/[*Bacteroides* + *Prevotella*]) within each population. The x-axis represents quantiles (from 0 to 1) to facilitate simultaneous plotting. The *black line* represents all the samples. The *green points* are from the Native African vs. African American Ou et al. study, the *yellow points* are from the Russian Urban vs Rural Tyakht et al. study, the *pink points* are from the Malawi, Venezuela, US Yatsunenko et al. study, the *red points* are from the Mixed Europe and Asia Arumugam et al. study, the *blue points* are from the European Arumugam et al. study, and the *light blue points* are from the NIH Human Microbiome Project study

To further understand groupings based on the abundance of *Prevotella*, we compared the *Prevotella* ratio (*Prevotella*/[*Bacteroides* + *Prevotella*]) for all samples in each study. Figure 2 illustrates the clear presence of samples across the full spectrum of relative abundances of *Prevotella* and *Bacteroides*, although different studies have varying numbers of samples in the intermediate ranges. Americans and Europeans, which make up the largest number of subjects, have fewer samples with intermediate *Prevotella* ratios, while studies containing more rural subjects have a greater number of intermediate samples. *Bacteroides* tends to be higher in Western populations, with a more even distribution of values across samples (Additional file 1: Figure S1), while *Prevotella* tends to have fewer intermediate values in the Western samples and more in Russian and rural samples (Additional file 6: Figure S2).

**Fig. 3:**
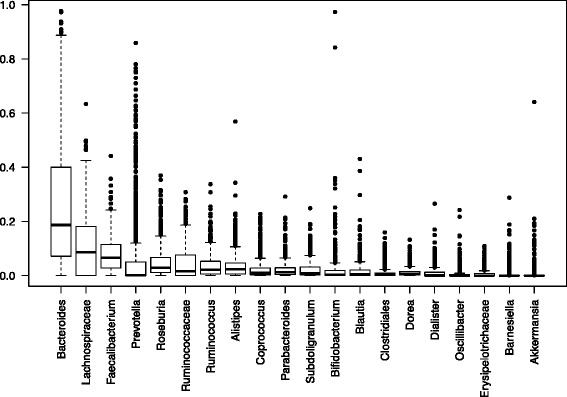
Boxplot of the top 20 taxa across all the studies. The *dark horizontal line* represents the mean relative abundance, and the *box* represents the bounds of the 25th and 75th percentiles

We compared the abundance distributions of *Prevotella* and *Bacteroides* with other common genera in the combined studies (Fig. 3 and Figure S11 in the Additional file 7 for the same boxplot without the Yatsunenko study). *Bacteroides*, *Prevotella*, *Lachnospiraceae*, *Faecalibacterium*, *Roseburia*, *Ruminococcus*, *Alistipes*, and *Coprococcus* are among the next most common genera across all studies. *Bacteroides*, *Prevotella*, and *Alistipes* are members of the order Bacteroidales, while the remaining taxa are all members of Clostridiales. In approximately 42 % of samples, *Bacteroides* has a relative abundance between 10 and 40 %, with more than 80 % of samples having a relative abundance greater than 5 %. *Prevotella* has a relative abundance between 10 and 40 % in 16 % of samples, but 75 % of samples have a relative abundance less than 5 %. While *Bacteroides* exhibits a higher proportion of intermediate abundances overall than does *Prevotella*, *Prevotella* shows a spectrum of abundances rather than a bimodal distribution as some have assumed, with more samples between 40 and 80 % than any taxon other than *Bacteroides*.

## Conclusions

Although several studies show some discrete clusters, most other studies have shown a continuous gradient of communities [2–7] that can change within an individual over time [6, 7], and no one has evaluated the taxonomic composition of the clusters across studies. The consistent body of evidence accumulating about the structure and role of the microbiome in human health demonstrates that standard microbiome methods are sufficient to compare across studies of the human gut. Here we analyze the combined microbiome data of five earlier studies and show that the gut microbiomes labeled as *Bacteroides* and *Prevotella* “enterotypes” do not represent consistent or predictable communities. No other bacterial taxa correlate with either of the two primary biomarkers across studies other than the taxa for which they are named. While the original study included a correlation analysis, the results represented the differences in their particular study populations, rather than a set of universal correlations. As was pointed out as early as 1896 by Pearson [20], proportions give rise to spurious correlations and our approach here relies on the study of proportions that eliminate the common denominators as suggested in [19].

Despite ongoing re-evaluation of the evidence, researchers have continued to use the term “enterotype” to describe differences between microbiome communities in study populations. The continued use of enterotype classifications is primarily a result of the ordination algorithms used to differentiate sets of samples based on the most distinguishing taxa. Of all the taxa in the combined studies, *Bacteroides* and *Prevotella* are the only two whose relative abundances frequently exceed 40 % (Fig. 3). Additionally, it is often the case that when *Bacteroides* is high in a sample, *Prevotella* will be low, and vice versa. These two taxa, therefore, contribute more than any others to the pairwise dissimilarity between samples. The various ordination method (PCoA, NMDS) plot samples with larger pairwise distances farther apart, and samples with smaller pairwise distances closer together. Because *Prevotella* and *Bacteroides* have the greatest range of values and tend to be inversely related, they contribute the most to the magnitude of pairwise distances, and therefore contribute the most to separation in ordination plots, leading to *Bacteroides*- and *Prevotella*-dominated gradients in ordination plots. Bacteroides abundance correlates significantly with the first axis, having a correlation of 0.93 (*p* value <10^-5). Prevotella abundance correlates significantly with the second axis having a negative correlation of −0.8 (*p* value <10^-4).

To illustrate how these *Bacteroides* and *Prevotella* abundances can drive ordination plots and that no other taxa were associated to the “enterotype” definitions, we compared ordination results with and without these two taxa (Fig. 1a, d, Additional file 8: Figure S4D, Additional file 9: Figure S5D, Additional file 3: Figure S7D, and Additional file 10: Figure S9D). We found that the remaining taxonomic-community members did not continue to separate these samples by microbial component classification. Ordination plots are not a means for defining underlying community characteristics and can only be part of the exploration of community structure rather than the endpoint of analysis. The dearth of any consistent features of these communities across independent studies is a reminder for us not to extrapolate from PCoA and NMDS plots to assumptions about the defining characteristics of the entire communities. In studies comparing only two very different populations (e.g., Western urban and non-Western rural), separation will likely remain even without *Bacteroides* and *Prevotella*, but we cannot extrapolate from any two populations to consistent clustering across a range of diverse populations when the two dominant taxa, *Bacteroides* and *Prevotella*, are removed. The combination of correlation results and ordinations with and without the dominant taxa can be used to compare *across* other studies to interpret clusters as driven by the most dominant taxa or by a deeper community structure.

We believe that using the term “enterotype” to describe gut communities is misleading because it implies a microbiome community type shared among samples with the same enterotype designation. We propose the use of the term “biomarker” to describe the dominant taxon of the community rather than “enterotypes” to describe gut microbiomes. Biomarkers serve as a measurable indicator of a biological state or environmental exposure [21]. In this regard, genera such as *Prevotella*, *Bacteroides*, and *Bifidobacterium* potentially serve as effective biomarkers of diet and lifestyle. Across the studies, *Prevotella* was associated with non-Western, rural communities, and a plant-based diet rich in polysaccharides and fiber [2, 8, 10, 11, 13, 21]. In each study sampling the gut microbiome of subjects from native or rural villages (Burkina Faso [8], South Africa [10], Venezuela and Malawi [11], and Russia [12]), *Prevotella* was commonly dominant in the adult samples. In these same studies, *Bacteroides*-dominated samples were more generally associated with samples from US and European subjects eating a Western diet, richer in protein and fat. Wu et al. [2] graphically illustrated correlations between *Bacteroides* and dietary amino acids and fats.

Beyond *Prevotella* and *Bacteroides*, additional taxa can also serve as biomarkers of diet or disease. *Bifidobacterium* was identified as a dominant bacterium in several studies [8, 11, 12], especially the gut microbiome of children [8, 11]. *Bifidobacterium* has specific adaptations that provide a competitive advantage for metabolizing the specific human milk oligosaccharides found in breast milk [9]. Various members of the phylum Firmicutes are associated with plant-derived fiber, amino acids, and fats. Firmicutes as a phylum, however, is unlikely to be an effective candidate for a biomarker, as it incorporates a diversity of genera. David et al. [22] report an increase in the bile-tolerant bacteria: *Alistipes*, *Bilophila*, and *Bacteroides* with an animal-based diet, while *Roseburia*, *Eubacterium*, and *Ruminococcus* were more abundant on a diet rich in plant polysaccharides. Some of these genera also have the potential to be biomarkers. Ou et al. [10] compared African Americans with a high risk of colon cancer and native Africans with only rare cases of colon cancer and suggested that *Bacteroides*, *Escherichia*, and *Acinetobacter* may be possible biomarkers for cancer. Baxter et al. [23] found correlations between *Bacteroides*, *Parabacteroides*, *Alistipes*, and *Akkermansia* with increased tumor burden and Clostridiales Cluster XIVa (which includes *Roseburia* and *Faecalibacterium*) with decreased tumor burden. *Fusobacterium* has also been associated with colon cancer [24]. The use of specific bacterial taxa to characterize the ecological roles of microbial community members was recently described by Trosvik and de Muinck [25]. They employed ecological concepts of keystone and foundation taxa, which complement their utility as biomarkers. Designation as keystone and foundation taxa requires knowledge of their causative role in a community, whereas biomarkers only need to demonstrate a correlation with diet, lifestyle, or disease state.

We have demonstrated that the “enterotypes” of the human gut microbiome do not represent recurrent microbial communities across the diversity of human populations, nor do they represent two distinguishable communities when *Bacteroides* and *Prevotella* are removed from the analysis. We propose that the dominant taxa associated with the original “enterotypes” hypothesis, *Bacteroides* and *Prevotella*, as well as other taxa such as *Bifidobacterium*, *Fusobacterium*, and various Clostridiales genera, are more accurately understood as biomarkers. The many correlative studies of the gut microbiome and associations with diet, environment, and disease can be made more useful by moving beyond basic ordination plots to rigorously assessing their sensitivity and specificity as biomarkers of lifestyle and disease in clinical subpopulations.

## Methods

### Data compilation

The data for this article were compiled from six previous studies. We used processed reads for each sample from the Yatsunenko et al. [11] study that were downloaded from the MG-RAST website (http://metagenomics.anl.gov) under the index qiime:850, MG-RAST IDs: 4489349.3 - 4489926.3. The reads were assigned taxonomy by comparison against the SILVA database [26] using GAST [27]. For all other studies, the data source was a table of taxonomic abundances for each sample. We received the Ou et al. [10] data directly from the authors. We downloaded the Human Microbiome Project (HMP) data from the VAMPS website [28] at the Marine Biological Laboratory (https://vamps.mbl.edu) under the name HMP_ST_v3v5. We downloaded the Arumugam et al. [1] datasets from the authors’ website (http://www.bork.embl.de/Docu/Arumugam_et_al_2011/downloads.html) under the “individual” subcategory of “Genus and phylum abundance tables of the three datasets.” We downloaded the Tyakht et al. [12] study data from the Russian Metagenome Project website (http://www.metagenome.ru/files/rus_met/).

The genera represented in the taxonomy tables were filtered to include only genera whose maximum relative abundance across all samples was greater than 0.0001 or 0.01 %. We also removed children under the age of 13 from the Yatsunenko et al. data as all other samples from the combined studies were adult, and we did not want to add childhood as a confounding variable. The summary of the data we received and the modifications we made for each statistical test can be found in supplemental materials (Additional file 4: Table S1). The taxonomic assignments made by each study were consistent with the NIH Human Microbiome Project and standard 16S databases with consistent taxonomic assignments, allowing the comparison of taxa across the various studies [29].

Lozupone et al. [30] analyzed the effectiveness of meta-study comparisons of human gut samples. Their reanalysis of the data, using OTUs (which they referred to as “species-level”), reproduced the results of the source publications. They specifically demonstrated that differences between Western and agrarian samples, along with age and geography, were ample to outweigh differences in study protocols. In some cases, however, they could discern study-specific clustering of samples from healthy Western guts, but the percent explained by their first two PCoA axes was only 12.5 % combined. We chose to use genus-level taxonomy rather than OTUs; the human gut taxa are well-represented in the 16S reference databases, the databases are consistent, and genus is a coarser-grained analysis, and therefore less sensitive than 97 % OTUs to differences in study design. We also note that the great abundance of research on the human gut microbiome using next-generation sequencing techniques is built on the presumption of consistent genus-level results across studies providing an increasing body of information on the nature of both healthy and dysbiotic human gut communities.

### Separation into microbial communities

We evaluated several methods for separating communities into their respective mixture components. *Bacteroides* is generally seen at a range of abundances including low, medium, and high abundances [2, 3, 10]. *Prevotella*, on the other hand, can have a bimodal behavior, with either a large relative abundance or almost none at all, especially in smaller studies [14, 15]. Roager et al. [16] found that the ratio of the logarithm of *Prevotella* to *Bacteroides* abundance generated a natural and distinct split between *Prevotella*- and *Bacteroides*-dominated microbial communities in their samples caused by the bimodal *Prevotella* distribution [5]. By compiling multiple studies, we uncovered a full range of *Prevotella* abundance, albeit the intermediate range abundances being less common (Additional file 11: Figure S3). The combined data therefore did not have a natural break in relative abundance values, and the log ratio method was ineffective. We chose instead to simply define *Prevotella*-dominated and *Bacteroides*-dominated samples as any sample in which either *Prevotella* or *Bacteroides* was the most abundant taxon. All remaining samples were classified together as “Other.” We introduced the *Prevotella* ratio as the ratio of *Prevotella* abundance to the combined abundance of *Prevotella* and *Bacteroides*, to measure the *Prevotella* abundance relative to *Bacteroides* in the samples (a value of 0 is no *Prevotella* and all *Bacteroides*, and a value of 1 is all *Prevotella* and no *Bacteroides*). This *Prevotella* ratio provides a continuous variable for measuring the spectrum of abundance values, rather than a binary value representing only the dominant taxon.

### Ordination

We performed both NMDS and PCoA with Bray-Curtis, Morisita-Horn, and Jensen-Shannon Divergence metrics (**ordinate** and **plot_ordination** functions in the **phyloseq** R package [18], and the **ellipse** package). See Figure S10 in Additional file 10 for the resulting ordination plots. Bray-Curtis was computed on the relative abundance. As expected, all methods provided similar results and we report only the PCoA results in the main paper (Fig. 1) and include the NMDS plots in the Additional files 12 and 13 (Figures S6 and S8 show the screeplots of the eigenvalues/percentage of variances explained). Plot points were labeled according to their most dominant taxa—*Bacteroides*, *Prevotella*, or other (Fig. 1a), by their population group within each study (Fig. 1c), or by their Prevotella ratio (defined above) (Fig. 1b). To illustrate the impact of the dominant taxon on the ordination of the microbial communities, we removed both *Prevotella* and *Bacteroides* rows in all the data and reran the ordination, without rescaling, to illustrate the change in separation (Fig. 1d).

### Correlations and differential abundance analyses

The number of DNA reads returned by next-generation sequencing for a bacterial sample does not reflect the absolute abundance of bacteria in the original sample. The total number of reads for each sample is effectively arbitrary and therefore represents only the relative not the actual abundance of DNA sequences. Unfortunately, relative abundance data are not statistically sufficient to accurately assess internal microbiome interactions [18], but as several of the studies only provided these proportions and not the original read numbers, we proceeded using relative abundance and took several precautions to avoid the biases this can induce. Since relative abundance values must always tally to 100 %, also known as compositional data, values within a sample are not independent and can lead to false negative correlations with parametric methods such as Pearson’s correlation. Nonparametric methods, such as Spearman’s correlation, are preferred in these types of analyses. We used the Spearman nonparametric method in R [31] (function cor.test) on the relative abundance matrices of the *Prevotella*-dominated and *Bacteroides*-dominated samples from all studies. We then confirmed our findings using SparCC which was developed by Friedman et al. [17] specifically to evaluate correlations within compositional data and remove false correlations through randomized bootstrap trials. We used SparCC with default parameters, generating 100 bootstrap files on all samples. For the studies for which sequence counts were available, the Malawi, Venezuela, and American study [11] and the HMP study of healthy Americans [15], we ran both SparCC [32] and nbinomTest of DESeq2 package [18] on the count data. To include datasets with only relative abundance data in the SparCC analysis, we multiplied the relative abundances by 10,000 to normalize sample counts to 10,000 reads, as per the recommendations of the SparCC authors. The choice of 10,000 meant that relative abundances as low as 0.0001 (0.01 %) were included. While converting from relative abundances to integer values of read counts would bias taxa that had a low abundance in samples that had been rarefied, none of the methods of the original studies included rarefaction. The correlations were run on the *Prevotella*-dominated and *Bacteroides*-dominated samples for each individual study independently and then on the compiled group of all studies. In all three methods, we defined statistically significant correlations as having a positive correlation coefficient and a multiple-experiments adjusted *p* value of less than or equal to 0.05 using the Benjamini-Hochberg algorithm (function p.adjust in R). We defined biologically significant correlations useful for developing a microbial community definition to be between community members with a mean abundance ≥0.02 (2 %) and a prevalence of 75 % (present in at least 75 % of the samples at ≥0.01 relative abundance. Bacteria with lower abundances or prevalences may perform important functions in the gut; they are not informative in a defining the overall community structure.

For the proportionality analysis, the Lovell et al. [19] proposed proportionality algorithm was applied to a table containing all the relative abundances for all the data sets with a modification to accommodate the large number of zero values in this very sparse data. Zero values were replaced by randomly generated uniform values between 10^(−8) and 2.10^(−8). Values that had a resulting phi value <0.01 were considered significant.

## Availability of supporting data

The data and the R script will be available at a permanent url for the html files associated to the article on publication (see the Rmd and html files have been deposited at the permanent url http://purl.stanford.edu/fs506ff9976).

## Additional files

Additional file 1: Figure S1. PCoA plots using the Bray distance metric with all the samples in the study colored by the origin of the data. (PDF 39 kb) Additional file 2: Figure S10. PCoA plot with all the samples except the Yatsunenko study colored by the most prominent taxon. A) Distance calculated using the Jensen-Shannon divergence (JSD) and B) distance calculated using Morisita-Horn. (PDF 17 kb) Additional file 3: Figure S7. PCoA plots using the Bray distance metric with only the Yatsunenko study. A) Samples colored by their most prominent taxon. If the sample is dominated neither by *Prevotella* nor *Bacteroides*, it is classified as other. B) Samples are colored by their value for the Prevotella ratio (relative abundance of *Prevotella*/[*Bacteroides* + *Prevotella*]) on a spectrum with red indicating no *Prevotella* and purple no *Bacteroides*. C) Samples are colored by population of origin. D) The Bray distance has been recalculated without the relative abundances of *Bacteroides* and *Prevotella*. Samples are colored by most prominent taxon in the original samples distributions (same as in plot A). (PDF 27 kb) Additional file 4: Table S1. Summary of the data received for each data set, as well as the format in which the data was used for the multivariate and proportionality analyses. (DOCX 81 kb) Additional file 5: RDATAand RMD.tar.gz is a zipped file of all the data and R markdown files to reproduce all the analyses done in the article and is the files can be found at : <http://purl.stanford.edu/fs506ff9976>. (ZIP 11504 kb) Additional file 6: Figure S2.
*Bacteroides* distributions within each population. The sample quantiles are represented on the x-axis and all samples are plotted on the same graph. The black line represents all the samples scaled together on the x-axis. The green points are from the Native African vs. African American Ou et al. study, the yellow points are from the Russian Urban vs Rural Tyakht et al. study, the pink points are from the Malawi, Venezuela, US Yatsunenko et al. study, the red points are from the Mixed Europe and Asia Arumugam et al. study, the blue points are from the European Arumugam et al. study, and the light blue points are from the NIH Human Microbiome Project study. (PDF 19 kb) Additional file 7: Figure S11. Boxplot of the top 20 taxa across studies not including the Yatsunenko study. The dark horizontal line represents the mean relative abundance and the box represents the bounds of the 25th and 75th percentiles. (PDF 25 kb) Additional file 8: Figure S4. NMDS plots using the Bray distance metric with all the samples except for the Yatsunenko study samples. A) Samples colored by their most prominent taxon. If the sample is dominated neither by *Prevotella* nor *Bacteroides*, it is classified as other. B) Samples are colored by their value for the Prevotella ratio (relative abundance of *Prevotella*/[*Bacteroides* + *Prevotella*]) on a spectrum with red indicating no *Prevotella* and purple no *Bacteroides*. C) Samples are colored by population of origin. D) The Bray distance has been recalculated without the relative abundances of *Bacteroides* and *Prevotella*. Samples are colored by most prominent taxon in the original samples distributions (same as in plot A). (PDF 46 kb) Additional file 9: Figure S5. A) Same PCoA plot using the Bray distance metric as in the main paper, except looking at the 3rd and 4th axis, with samples colored by their most prominent taxa. B) Same MDS plot as in A, but with samples colored based on their value for the Prevotella ratio on a spectrum with red indicating no *Prevotella* and purple no *Bacteroides*. C) Same PCoA plot with samples colored based on population of origin. D) PCoA plot using the Bray distance metric with the *Bacteroides* and *Prevotella* relative abundances taken out. Colored by most prominent taxa in the samples before the removal of *Prevotella* and *Bacteroides*. (PDF 47 kb) Additional file 10: Figure S9. NMDS plots using the Bray distance metric with only the Yatsunenko study. A) Samples colored by their most prominent taxon. If the sample is dominated neither by *Prevotella* nor *Bacteroides*, it is classified as other. B) Samples are colored by their value for the Prevotella ratio (relative abundance of *Prevotella*/[*Bacteroides* + *Prevotella*]) on a spectrum with red indicating no *Prevotella* and purple no *Bacteroides*. C) Samples are colored by population of origin. D) The Bray distance has been recalculated without the relative abundances of *Bacteroides* and *Prevotella*. Samples are colored by most prominent taxon in the original samples distributions (same as in plot A). (PDF 28 kb) Additional file 11: Figure S3.
*Prevotella* distributions within each population. The x-axis represents the sample quantiles and all the samples are plotted on the same graph. The black line represents all the samples scaled together on the x-axis. The green points are from the Native African vs. African American Ou et al. study, the yellow points are from the Russian Urban vs Rural Tyakht et al. study, the pink points are from the Malawi, Venezuela, US Yatsunenko et al. study, the red points are from the Mixed Europe and Asia Arumugam et al. study, the dark blue points are from the European Arumugam et al. study, and the light blue points are from the NIH Human Microbiome Project study. (PDF 16 kb) Additional file 12: Figure S6. Relative variances explained by the PCoA axes from the PCoA analyses. A) PCoA variances explained by the axes used in the Fig. 1a, b and c and Figure S5ABC. B) PCoA variances axes used in the adjusted sample plots in Fig. 1d and Figure S5D, which had its *Bacteroides* and *Prevotella* relative abundances removed. (PDF 4 kb) Additional file 13: Figure S8. Relative variances explained by the PCoA axes from the PCoA analyses A) Relative variances explained by the PCoA axes used in the Additional file 7: Figure S7ABC. B) Relative variances explained by the PCoA axes used in the adjusted sample plot in Additional file 7: Figure S7D, which had its *Bacteroides* and *Prevotella* relative abundances removed. (PDF 4 kb)

## Footnotes

1 Combining “enteric” from the Greek word for intestine, and the English word “type” meaning category. Here, we use the term as originally intended.

2 PCoA and MDS are abbreviations for Principal Coordinates Analysis and Multidimensional Scaling, two names for the same ordination method which aims to provide low dimensional Euclidean representations of multivariate data for which a specific distance has been measured.

3 NMDS designates non-metric multidimensional scaling, a non-parametric version of MDS that aims to provide a Euclidean graphical representation of the data points where the distances between points have the same order as those in the original data.
