# Supplementary figures and images for: Interpreting Prevotella and Bacteroides as biomarkers of diet and lifestyle
Source: Microbiome. 2016 Apr 12;4:15. doi: 10.1186/s40168-016-0160-7 (PMC4828855; doi:10.1186/s40168-016-0160-7)

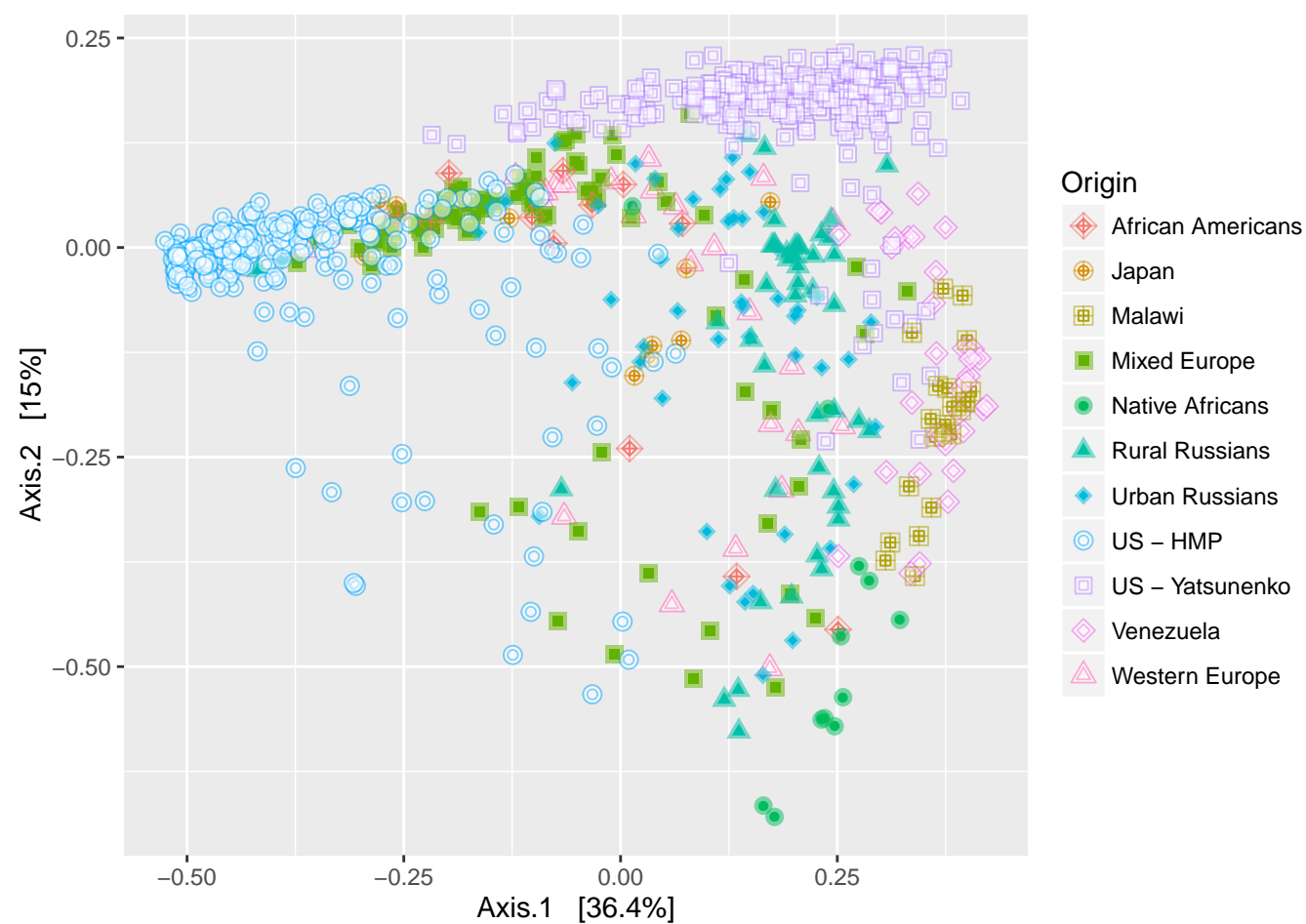

Supplement: Additional file 1: Figure S1. — PCoA plots using the Bray distance metric with all the samples in the study colored by the origin of the data. (PDF 39 kb) [file 40168_2016_160_MOESM1_ESM.pdf]

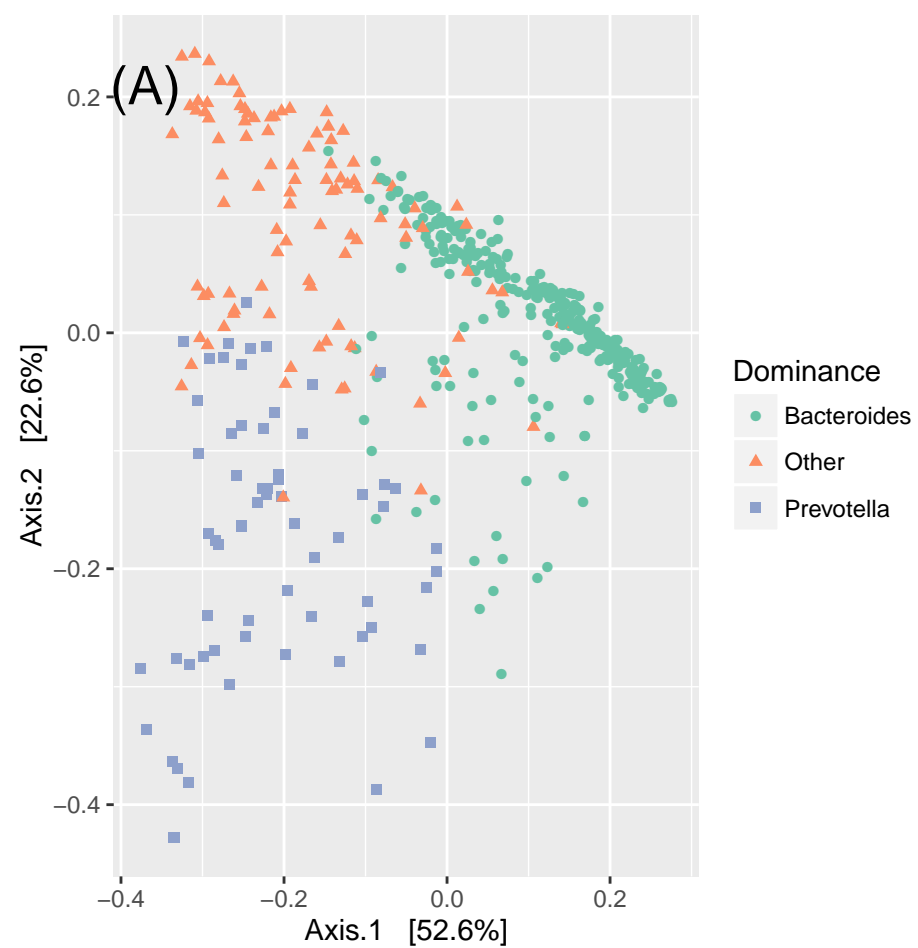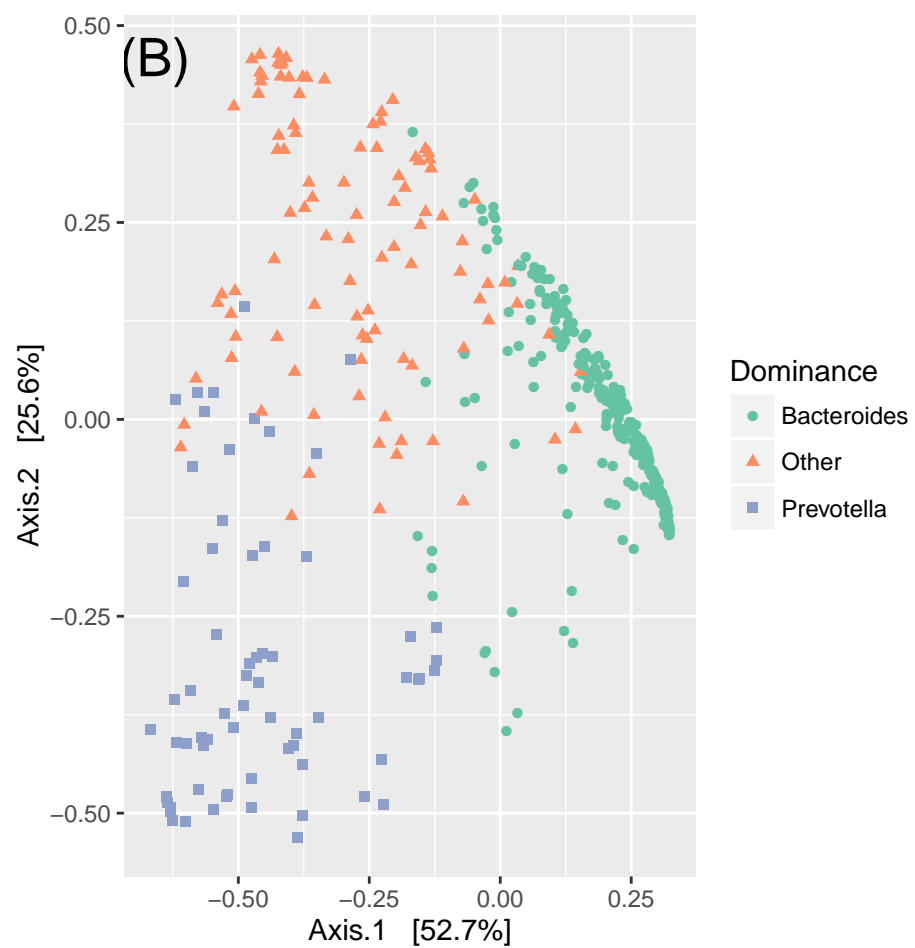

Supplement: Additional file 2: Figure S10. — PCoA plot with all the samples except the Yatsunenko study colored by the most prominent taxon. A) Distance calculated using the Jensen-Shannon divergence (JSD) and B) distance calculated using Morisita-Horn. (PDF 17 kb) [file 40168_2016_160_MOESM2_ESM.pdf]

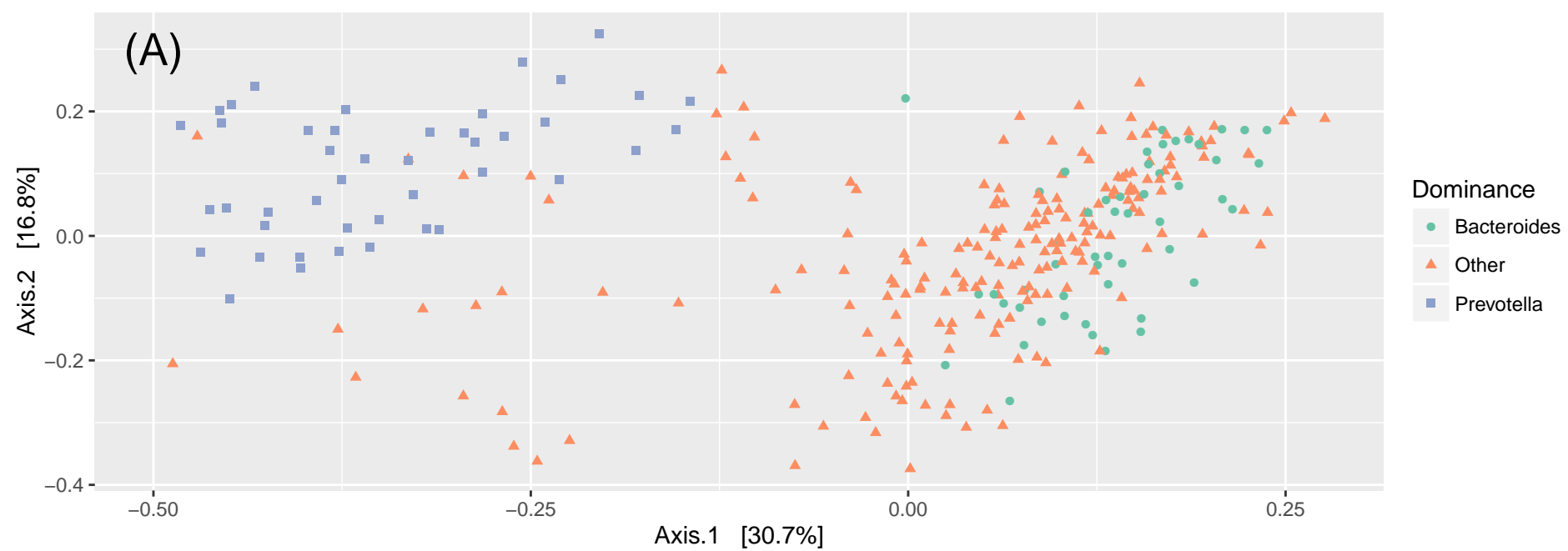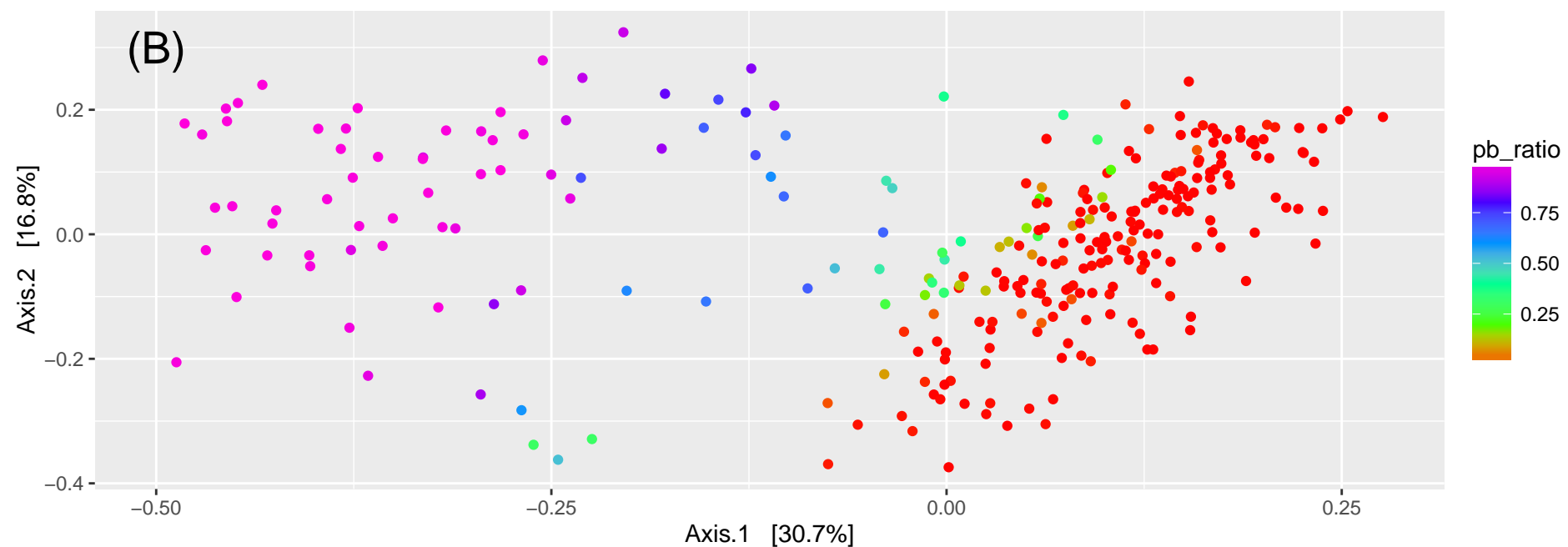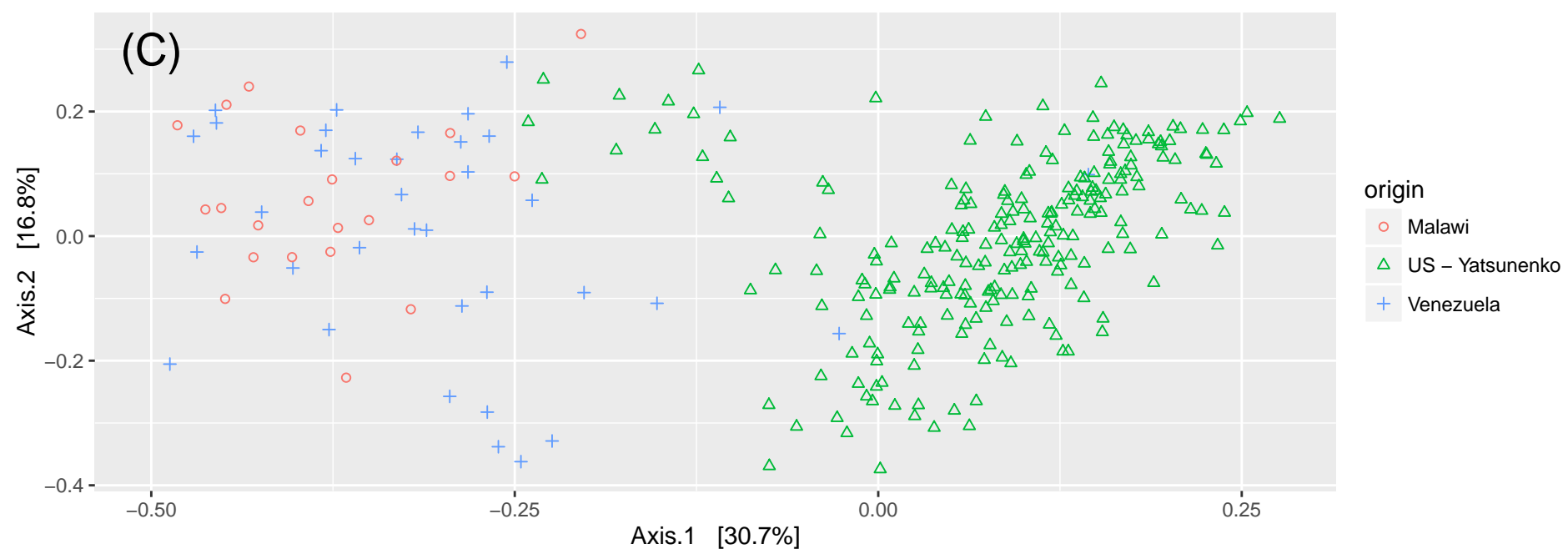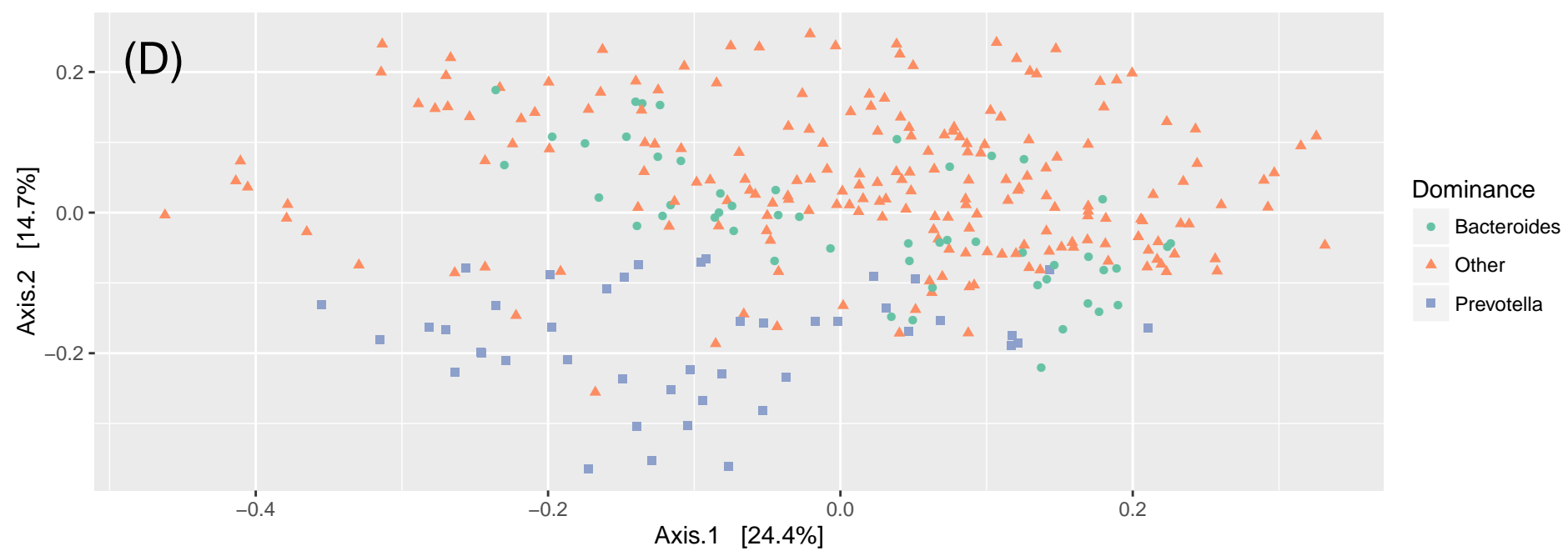

Supplement: Additional file 3: Figure S7. — PCoA plots using the Bray distance metric with only the Yatsunenko study. A) Samples colored by their most prominent taxon. If the sample is dominated neither by Prevotella nor Bacteroides, it is classified as other. B) Samples are colored by their value for the Prevotella ratio (relative abundance of Prevotella/[Bacteroides + Prevotella]) on a spectrum with red indicating no Prevotella and purple no Bacteroides. C) Samples are colored by population of origin. D) The Bray distance has been recalculated without the relative abundances of Bacteroides and Prevotella. Samples are colored by most prominent taxon in the original samples distributions (same as in plot A). (PDF 27 kb) [file 40168_2016_160_MOESM3_ESM.pdf]

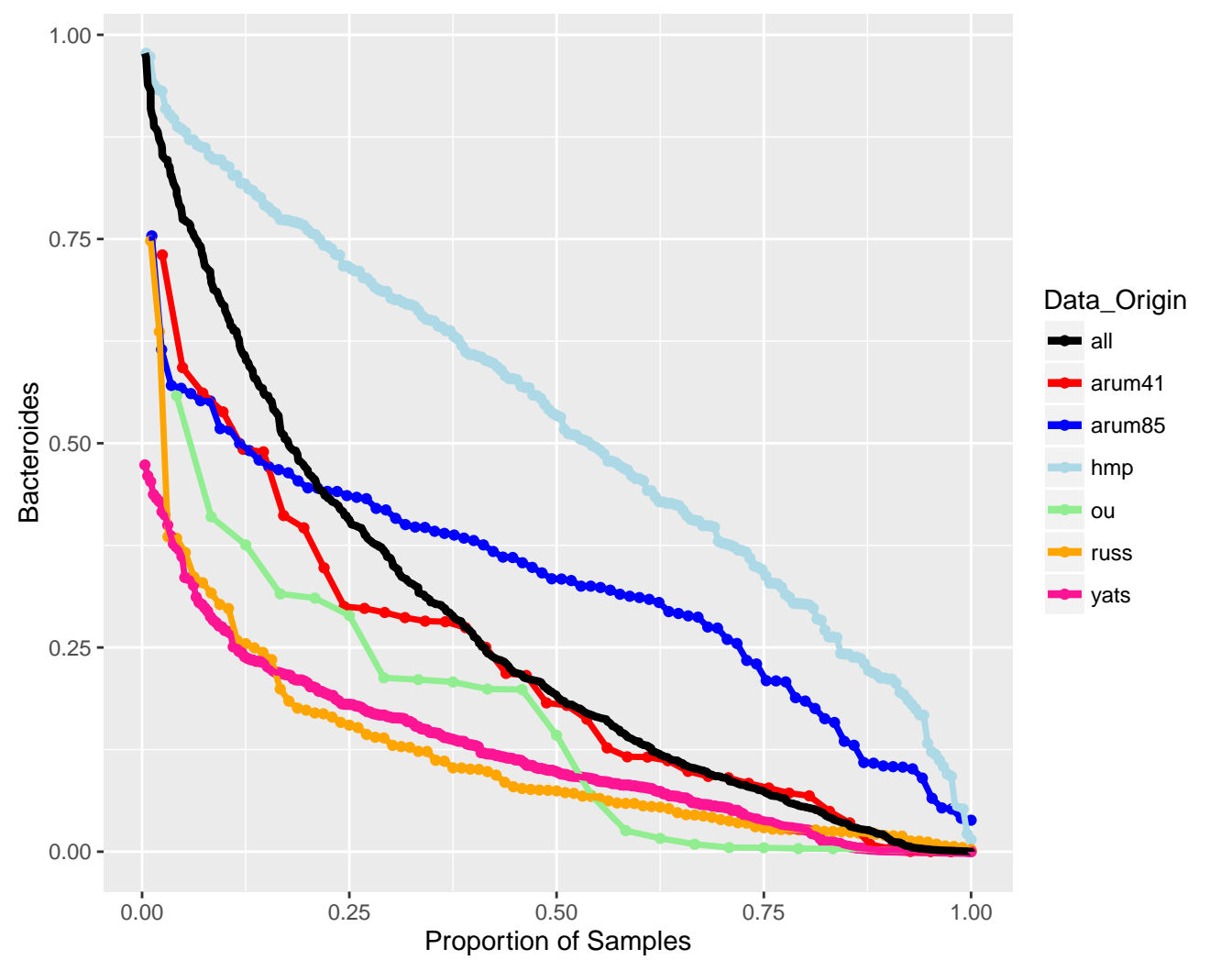

Supplement: Additional file 6: Figure S2. — Bacteroides distributions within each population. The sample quantiles are represented on the x-axis and all samples are plotted on the same graph. The black line represents all the samples scaled together on the x-axis. The green points are from the Native African vs. African American Ou et al. study, the yellow points are from the Russian Urban vs Rural Tyakht et al. study, the pink points are from the Malawi, Venezuela, US Yatsunenko et al. study, the red points are from the Mixed Europe and Asia Arumugam et al. study, the blue points are from the European Arumugam et al. study, and the light blue points are from the NIH Human Microbiome Project study. (PDF 19 kb) [file 40168_2016_160_MOESM6_ESM.pdf]

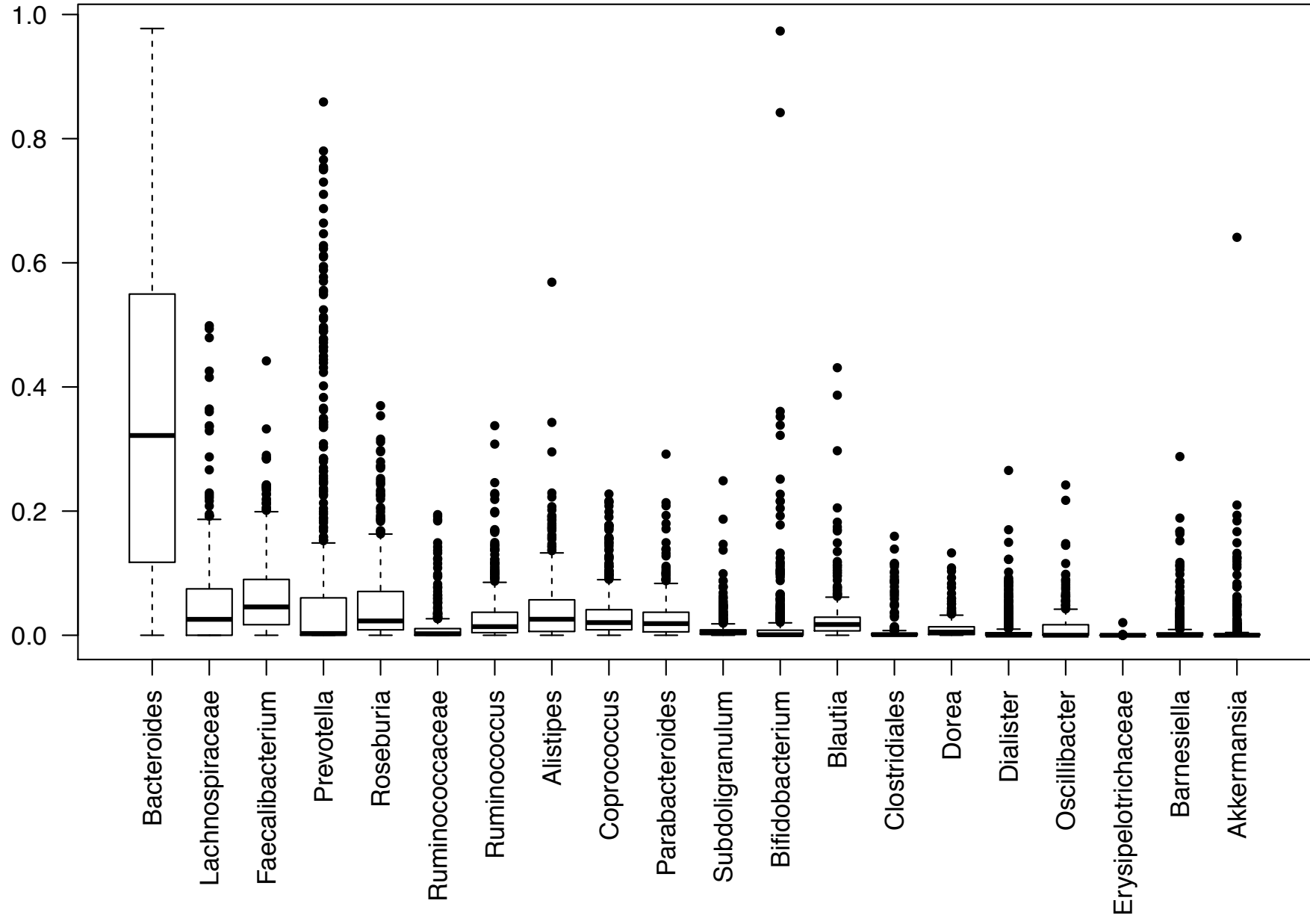

Supplement: Additional file 7: Figure S11. — Boxplot of the top 20 taxa across studies not including the Yatsunenko study. The dark horizontal line represents the mean relative abundance and the box represents the bounds of the 25th and 75th percentiles. (PDF 25 kb) [file 40168_2016_160_MOESM7_ESM.pdf]

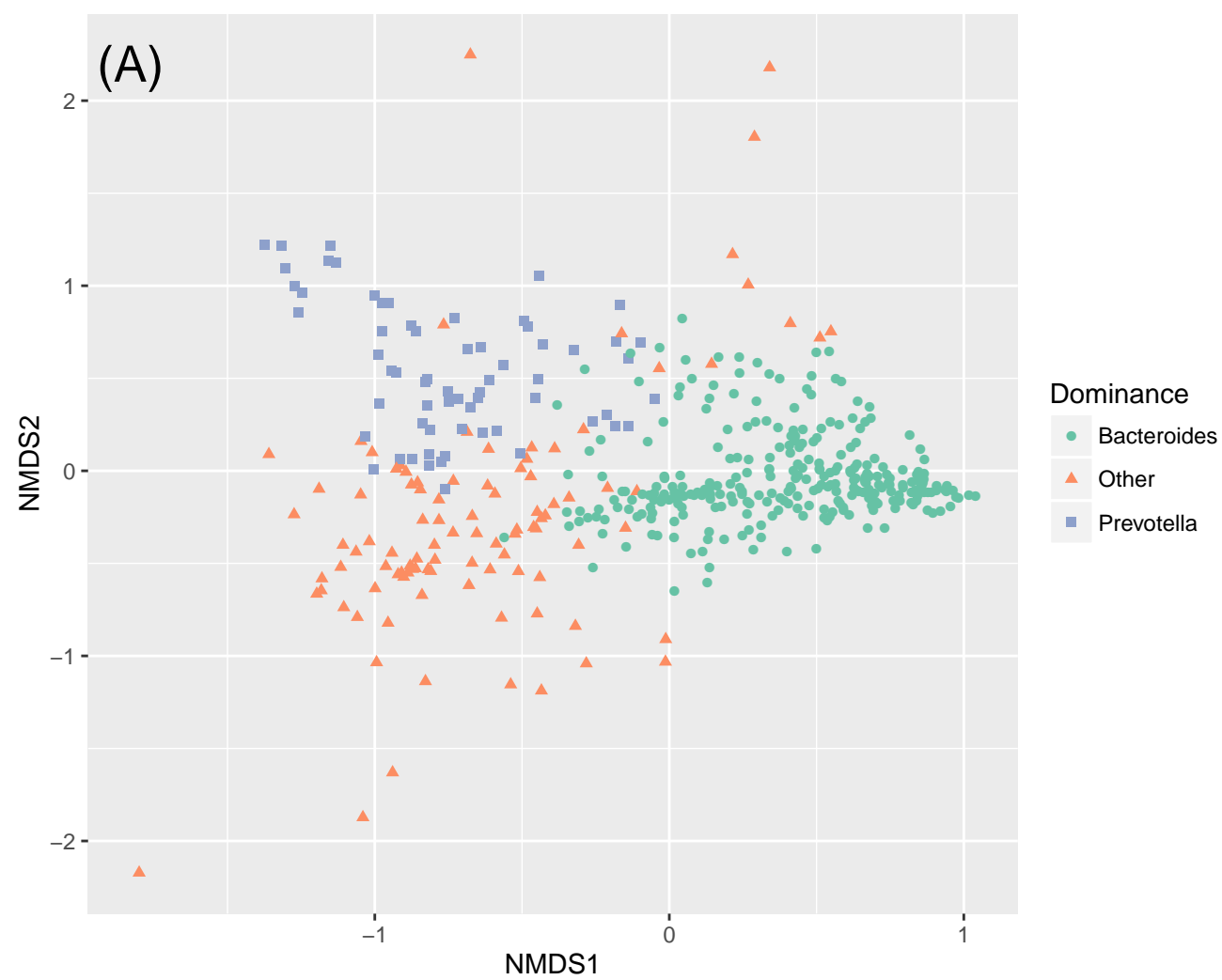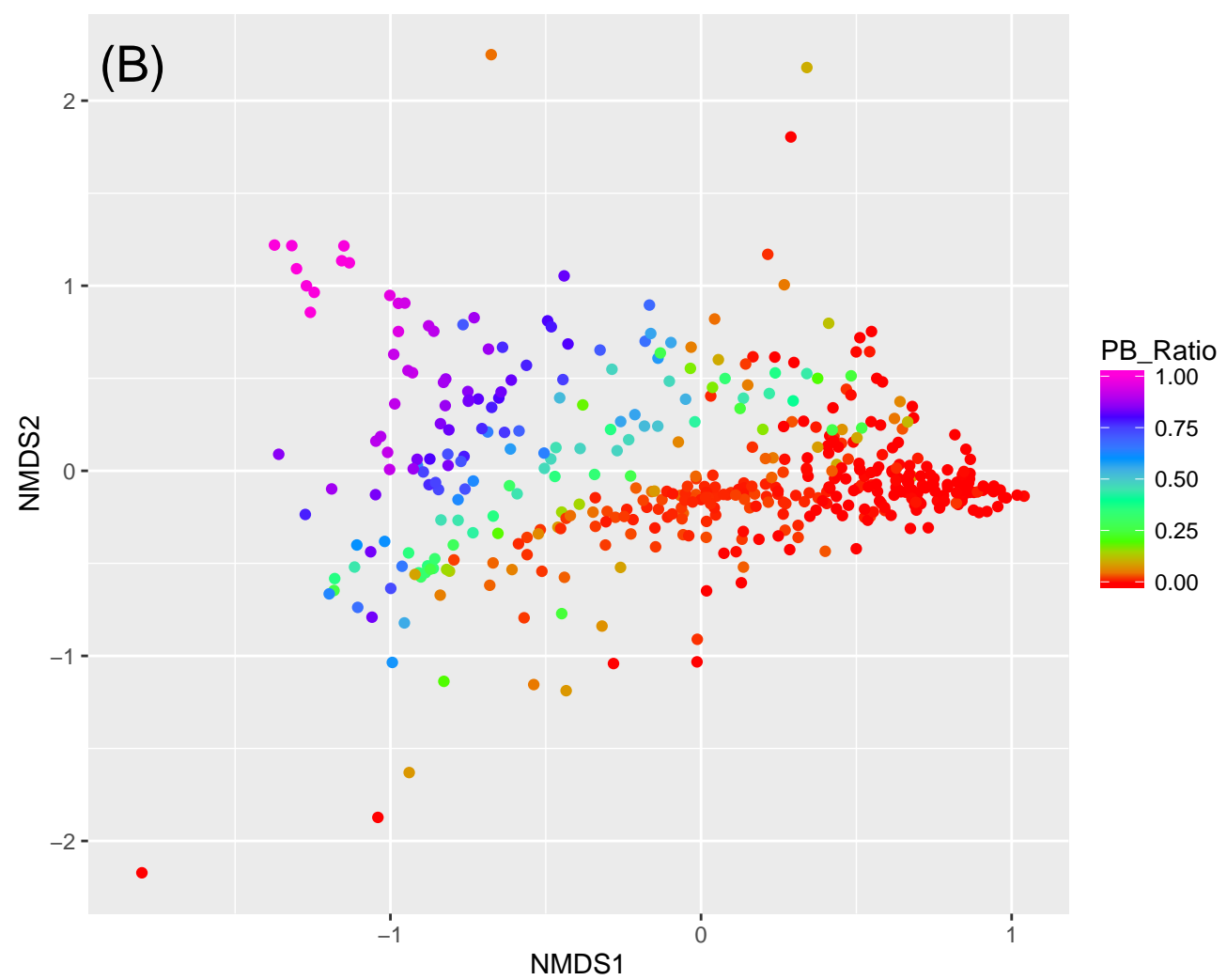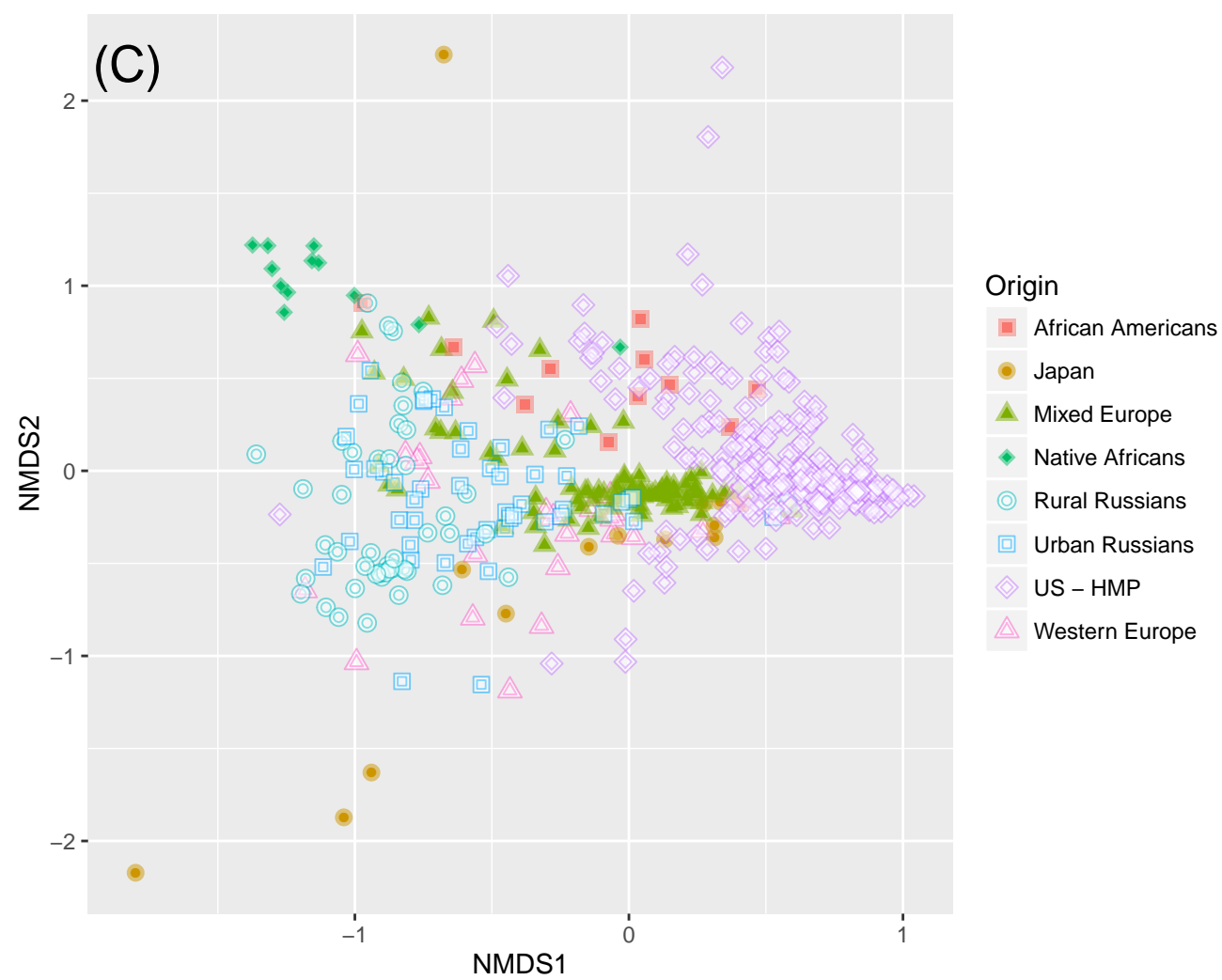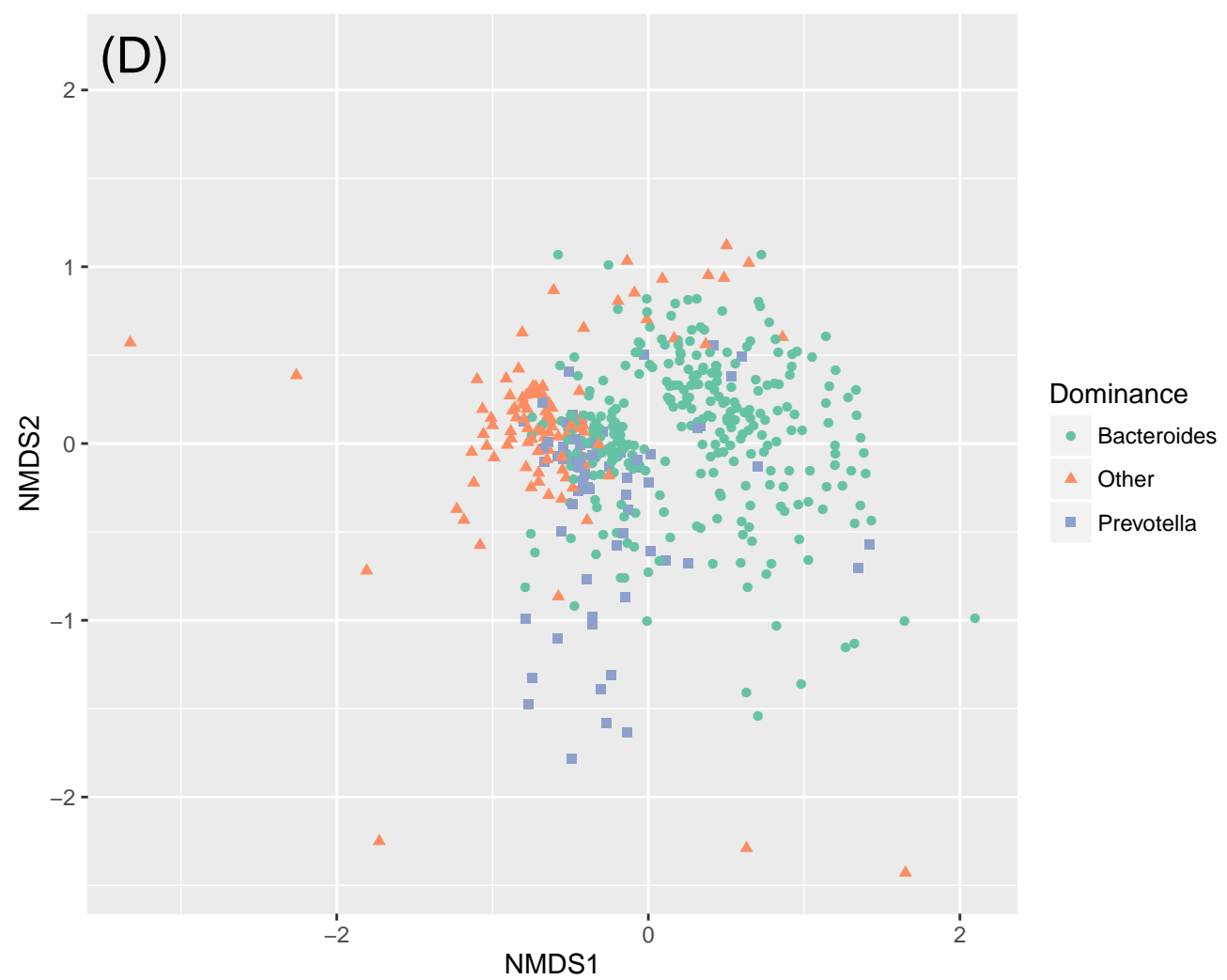

Supplement: Additional file 8: Figure S4. — NMDS plots using the Bray distance metric with all the samples except for the Yatsunenko study samples. A) Samples colored by their most prominent taxon. If the sample is dominated neither by Prevotella nor Bacteroides, it is classified as other. B) Samples are colored by their value for the Prevotella ratio (relative abundance of Prevotella/[Bacteroides + Prevotella]) on a spectrum with red indicating no Prevotella and purple no Bacteroides. C) Samples are colored by population of origin. D) The Bray distance has been recalculated without the relative abundances of Bacteroides and Prevotella. Samples are colored by most prominent taxon in the original samples distributions (same as in plot A). (PDF 46 kb) [file 40168_2016_160_MOESM8_ESM.pdf]

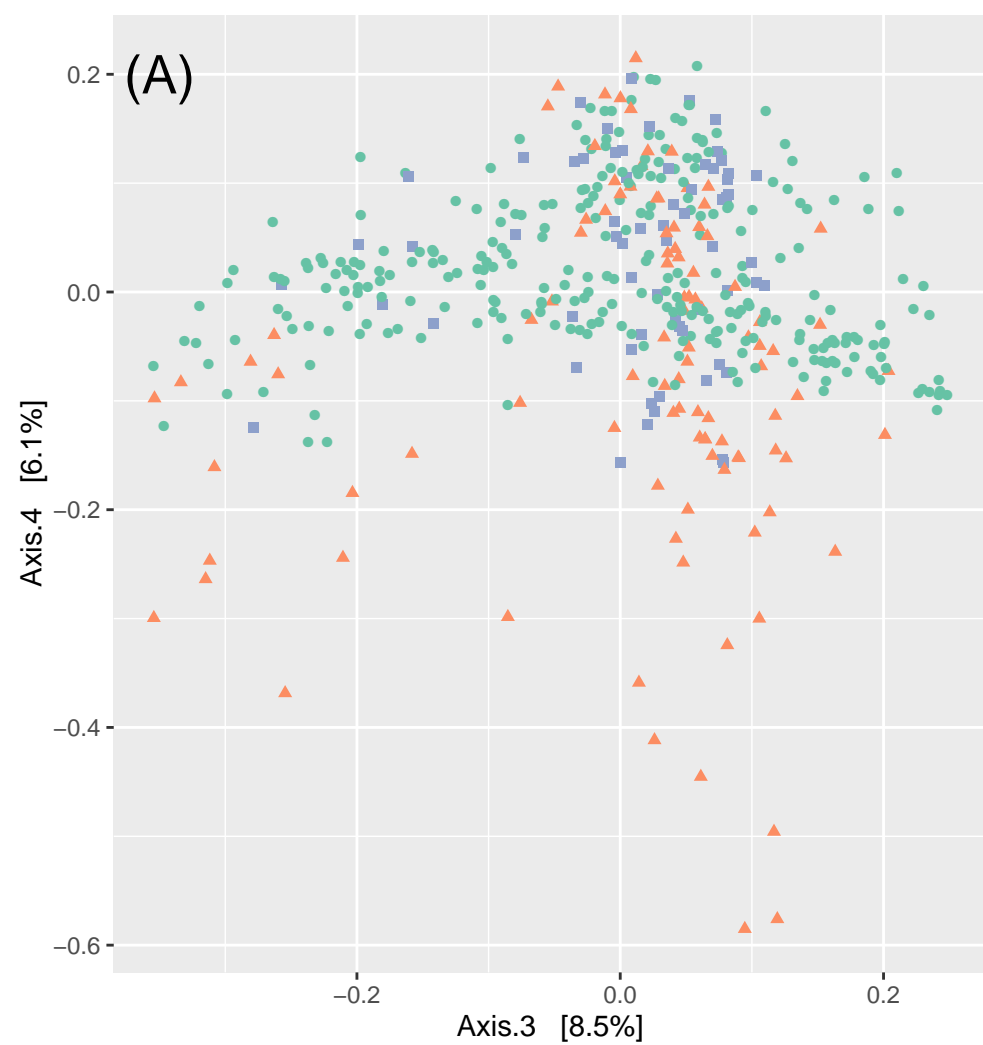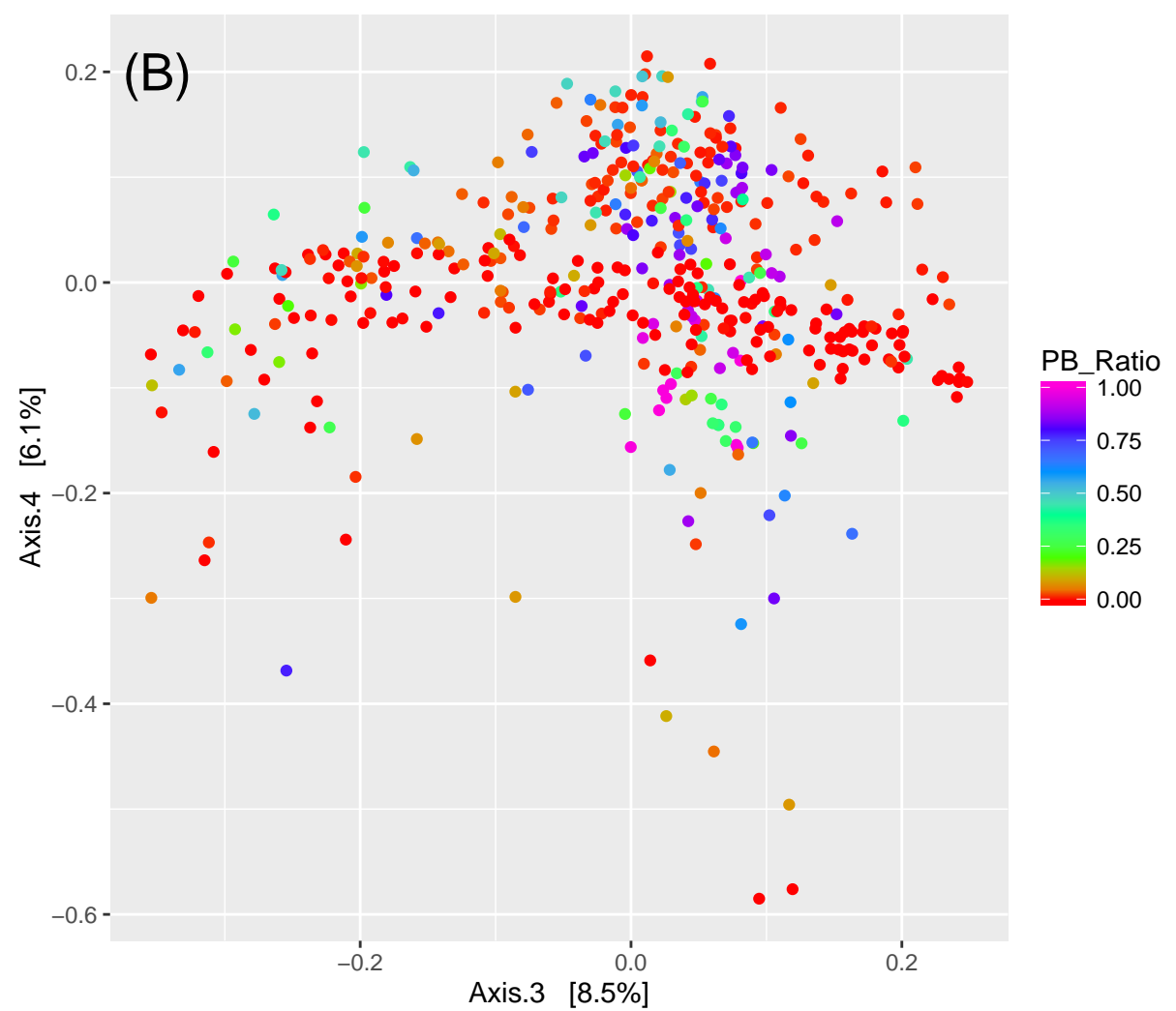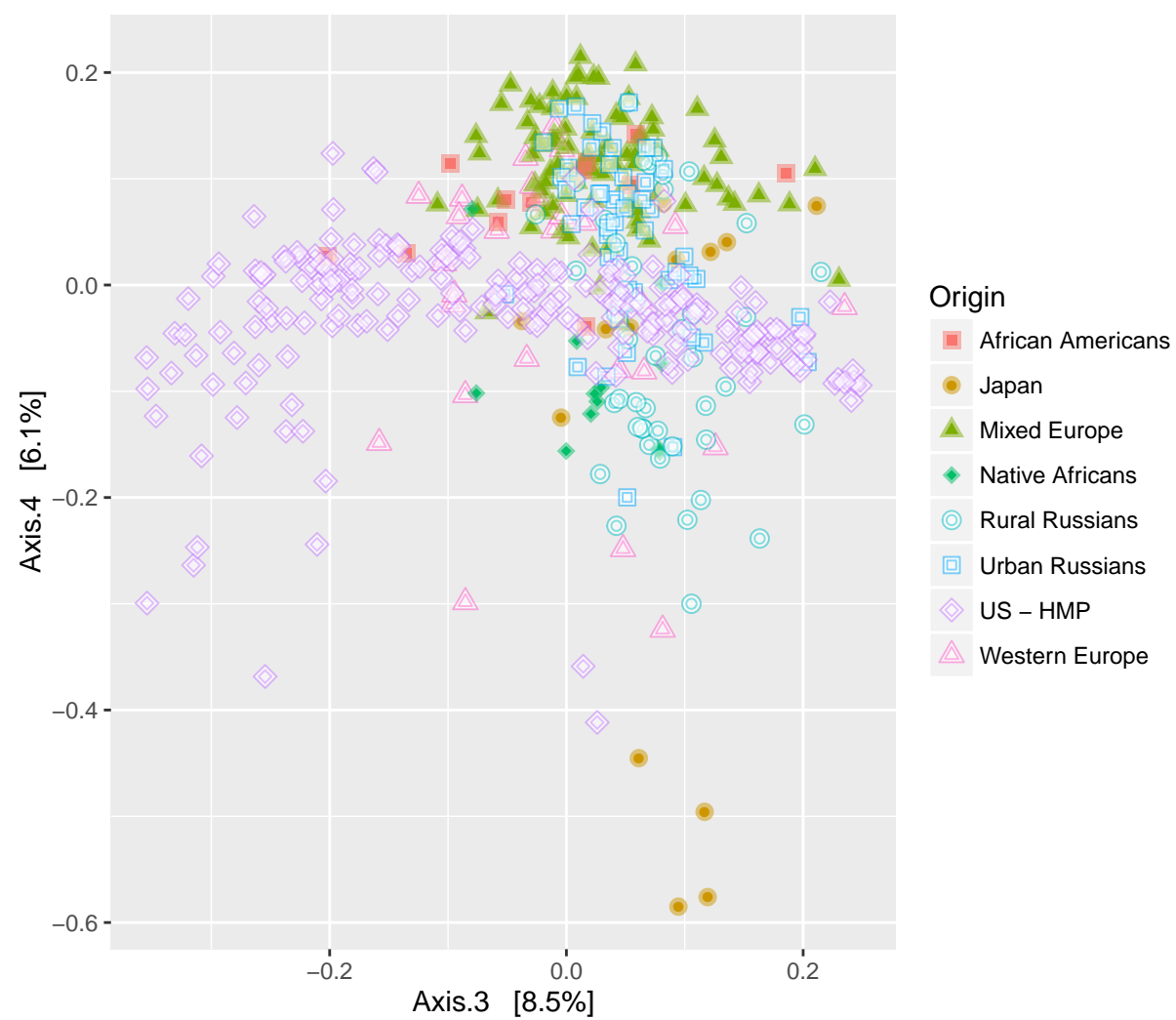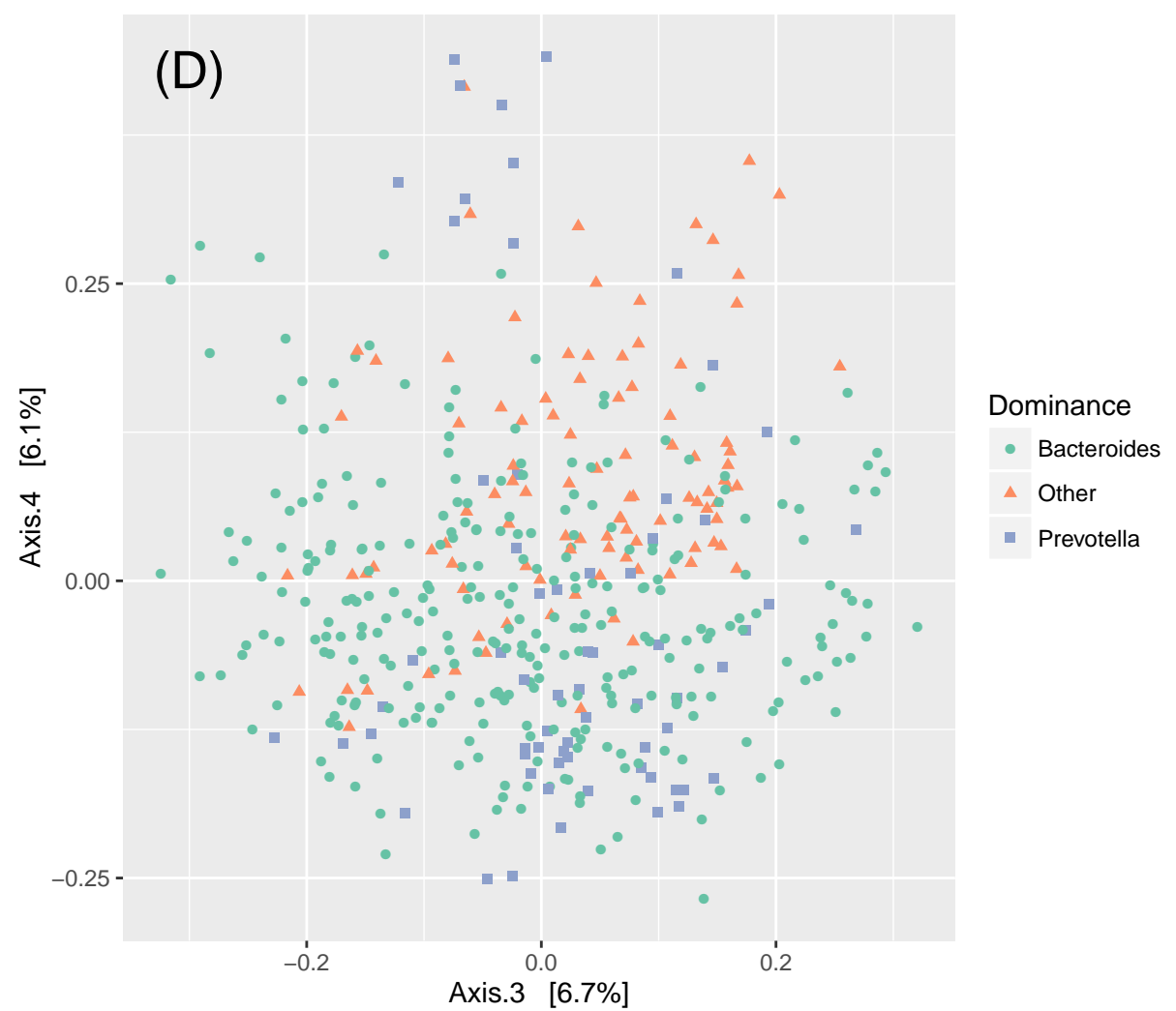

Supplement: Additional file 9: Figure S5. — A) Same PCoA plot using the Bray distance metric as in the main paper, except looking at the 3rd and 4th axis, with samples colored by their most prominent taxa. B) Same MDS plot as in A, but with samples colored based on their value for the Prevotella ratio on a spectrum with red indicating no Prevotella and purple no Bacteroides. C) Same PCoA plot with samples colored based on population of origin. D) PCoA plot using the Bray distance metric with the Bacteroides and Prevotella relative abundances taken out. Colored by most prominent taxa in the samples before the removal of Prevotella and Bacteroides. (PDF 47 kb) [file 40168_2016_160_MOESM9_ESM.pdf]

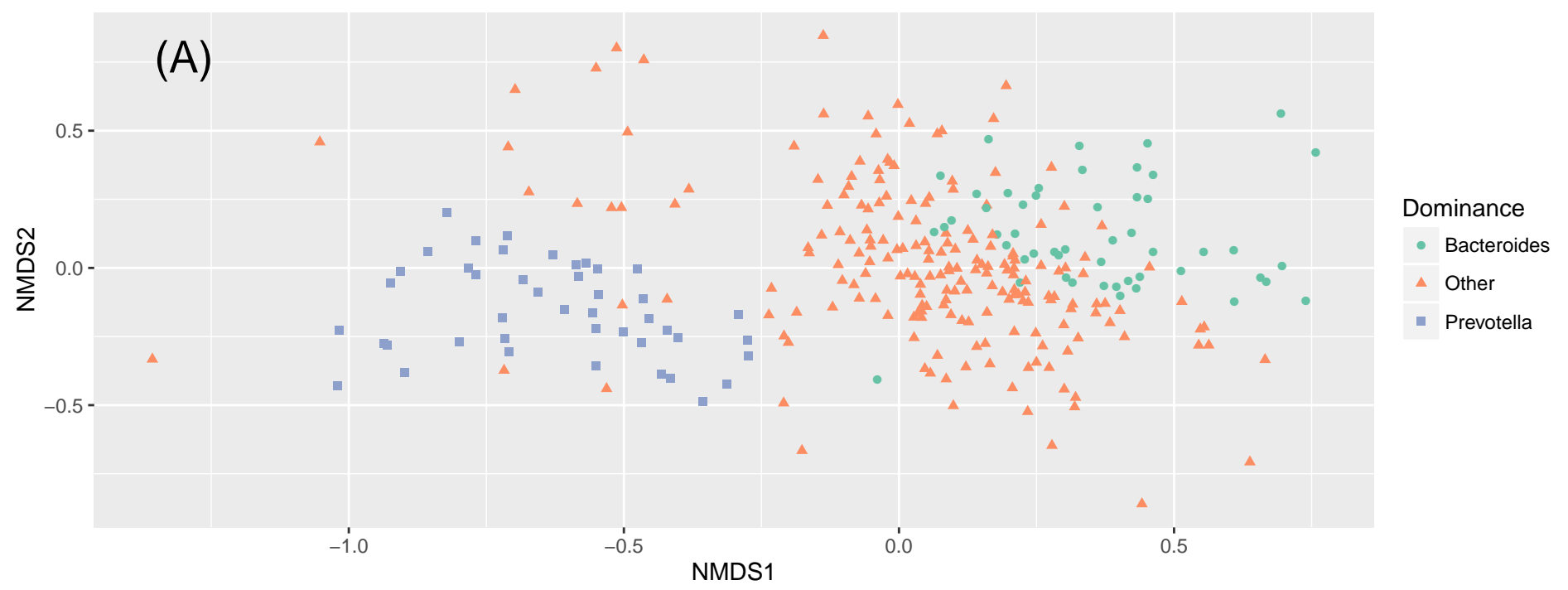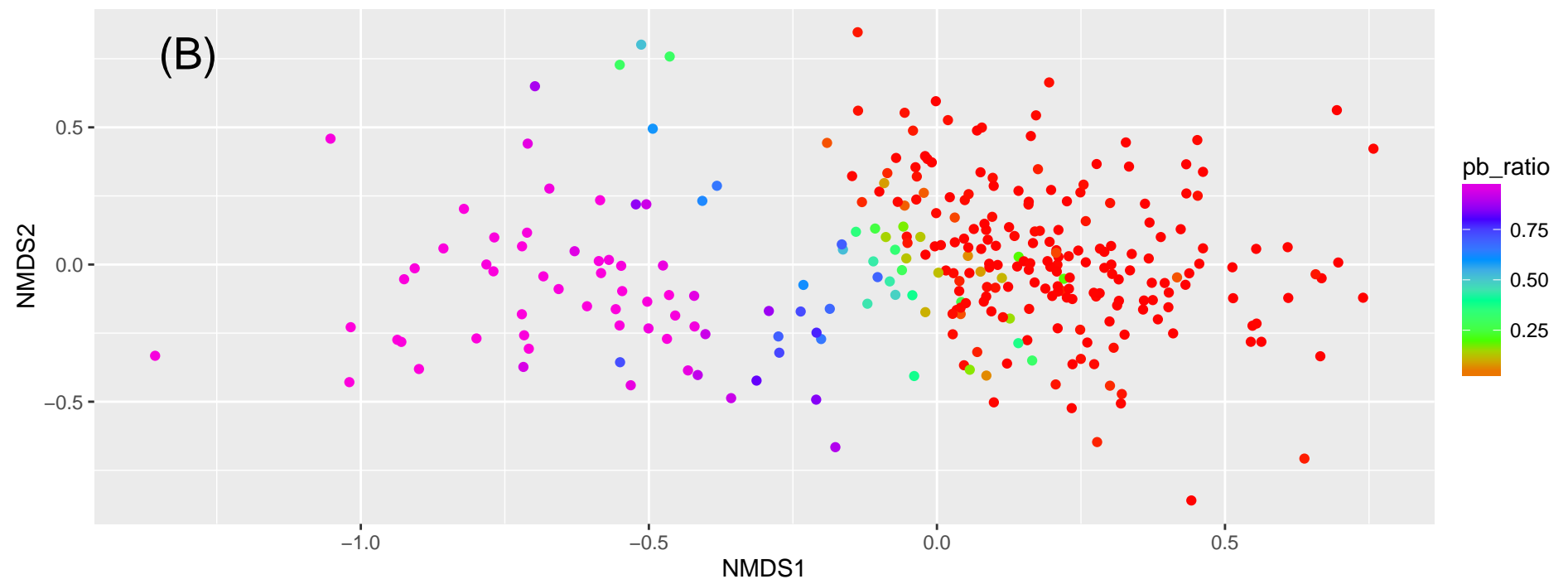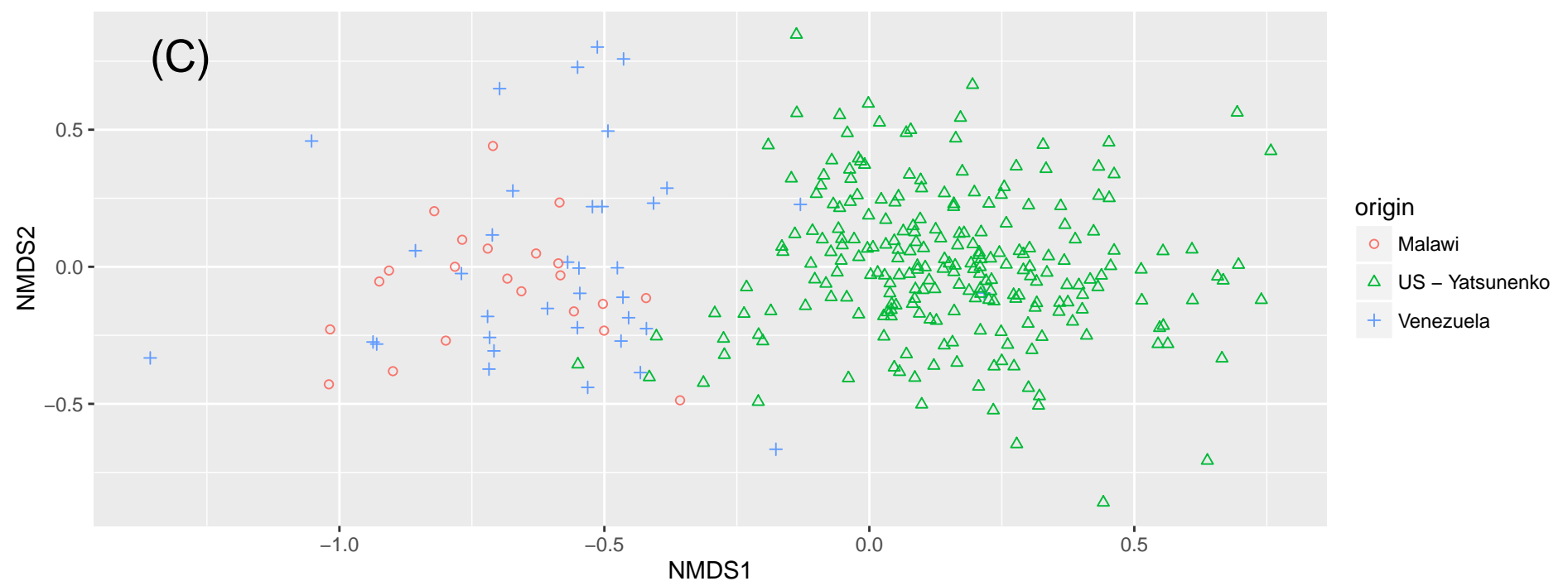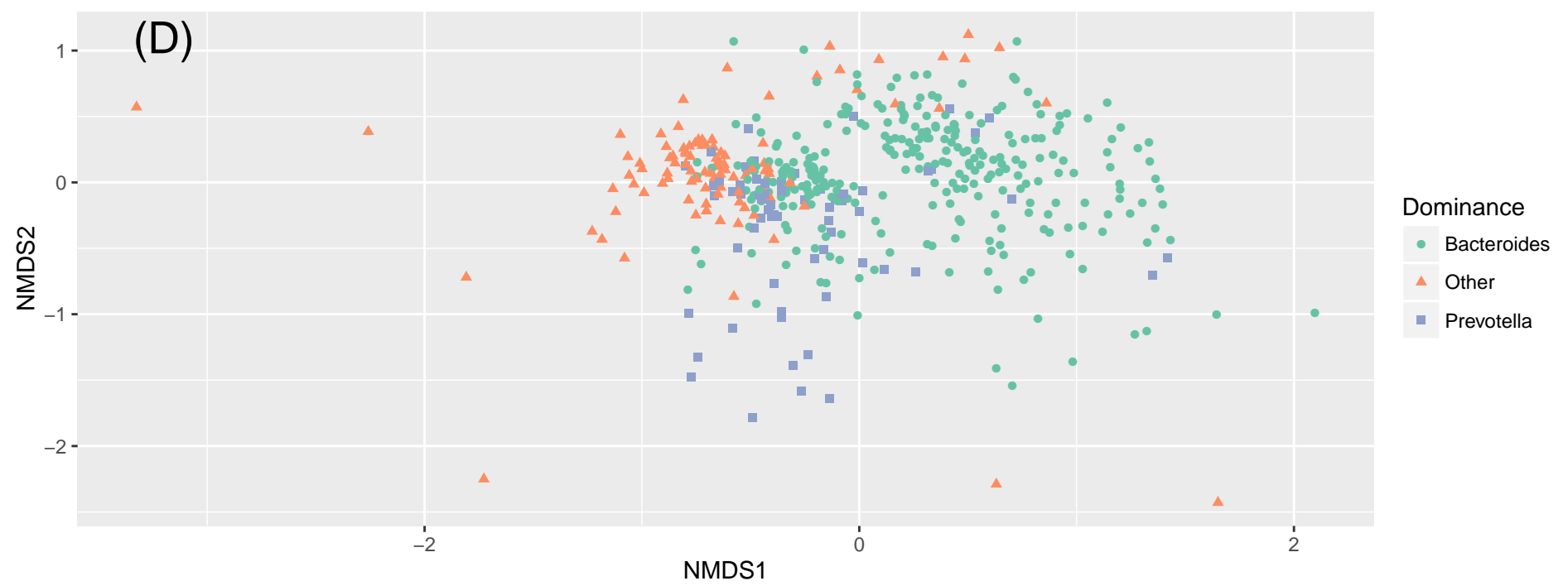

Supplement: Additional file 10: Figure S9. — NMDS plots using the Bray distance metric with only the Yatsunenko study. A) Samples colored by their most prominent taxon. If the sample is dominated neither by Prevotella nor Bacteroides, it is classified as other. B) Samples are colored by their value for the Prevotella ratio (relative abundance of Prevotella/[Bacteroides + Prevotella]) on a spectrum with red indicating no Prevotella and purple no Bacteroides. C) Samples are colored by population of origin. D) The Bray distance has been recalculated without the relative abundances of Bacteroides and Prevotella. Samples are colored by most prominent taxon in the original samples distributions (same as in plot A). (PDF 28 kb) [file 40168_2016_160_MOESM10_ESM.pdf]

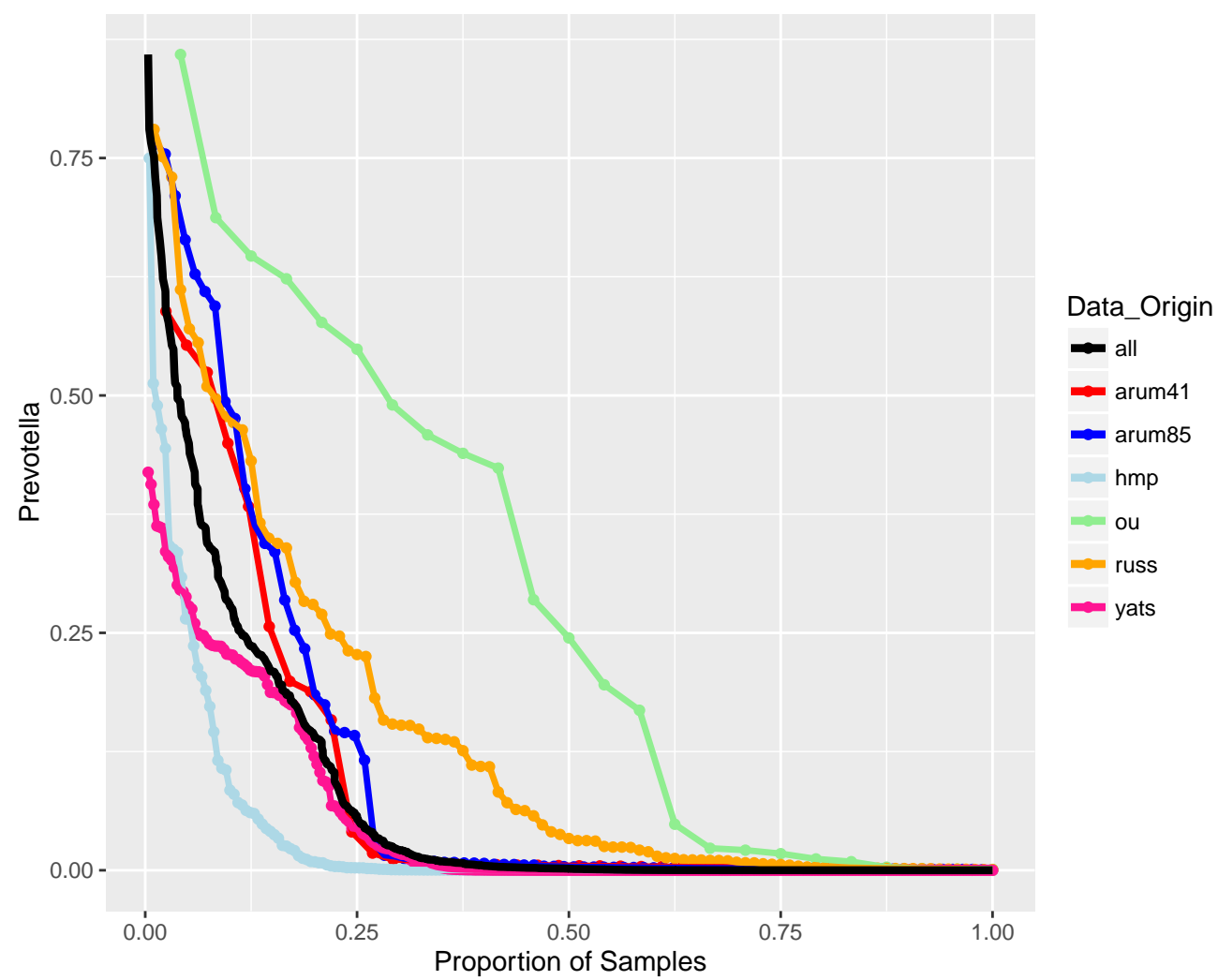

Supplement: Additional file 11: Figure S3. — Prevotella distributions within each population. The x-axis represents the sample quantiles and all the samples are plotted on the same graph. The black line represents all the samples scaled together on the x-axis. The green points are from the Native African vs. African American Ou et al. study, the yellow points are from the Russian Urban vs Rural Tyakht et al. study, the pink points are from the Malawi, Venezuela, US Yatsunenko et al. study, the red points are from the Mixed Europe and Asia Arumugam et al. study, the dark blue points are from the European Arumugam et al. study, and the light blue points are from the NIH Human Microbiome Project study. (PDF 16 kb) [file 40168_2016_160_MOESM11_ESM.pdf]

(A)

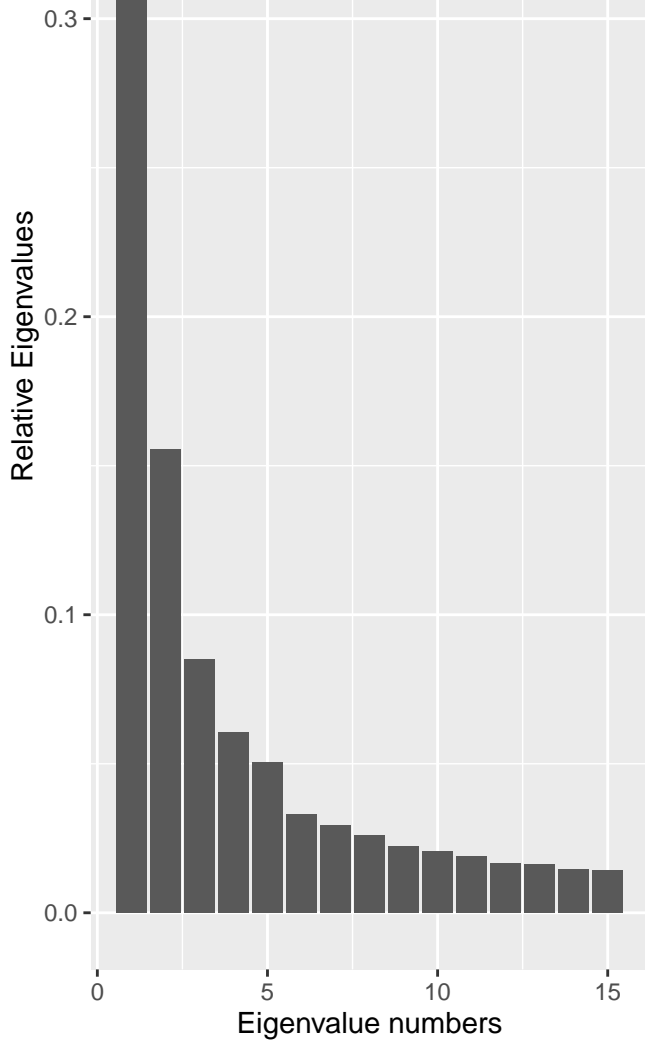

(B)

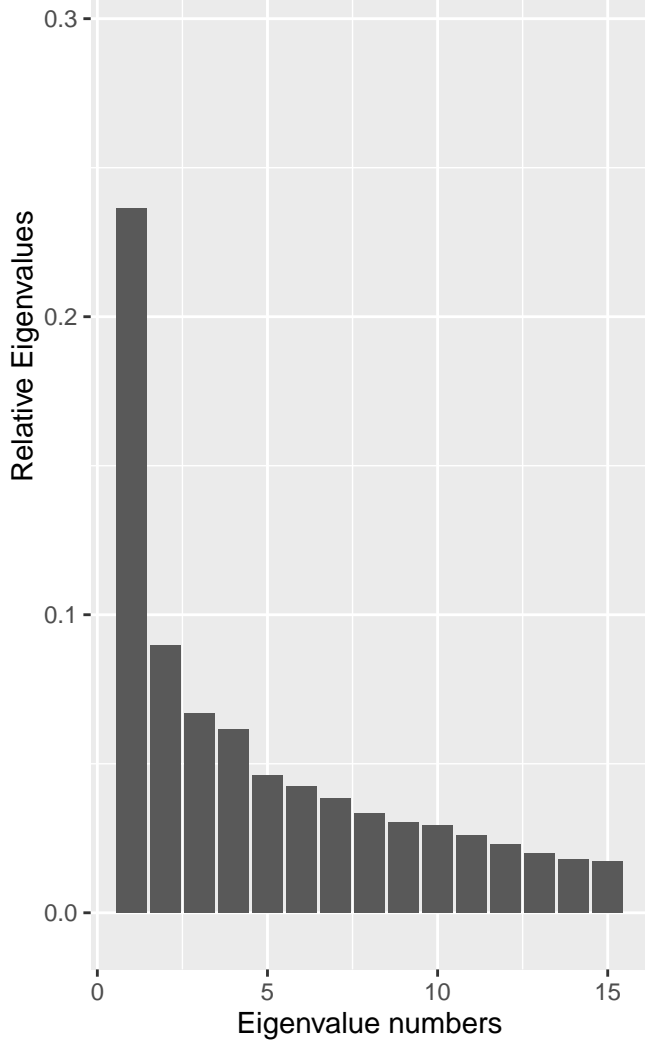

Supplement: Additional file 12: Figure S6. — Relative variances explained by the PCoA axes from the PCoA analyses. A) PCoA variances explained by the axes used in the Fig. 1a, b and c and Figure S5ABC. B) PCoA variances axes used in the adjusted sample plots in Fig. 1d and Figure S5D, which had its Bacteroides and Prevotella relative abundances removed. (PDF 4 kb) [file 40168_2016_160_MOESM12_ESM.pdf]

(A)

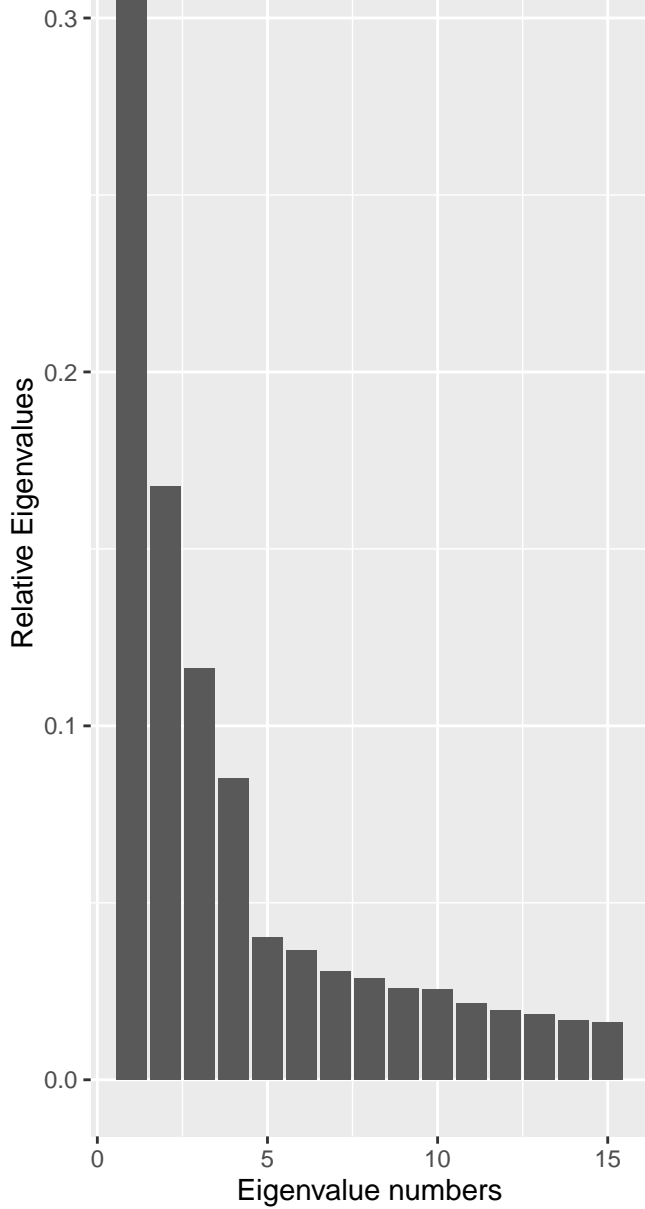

(B)

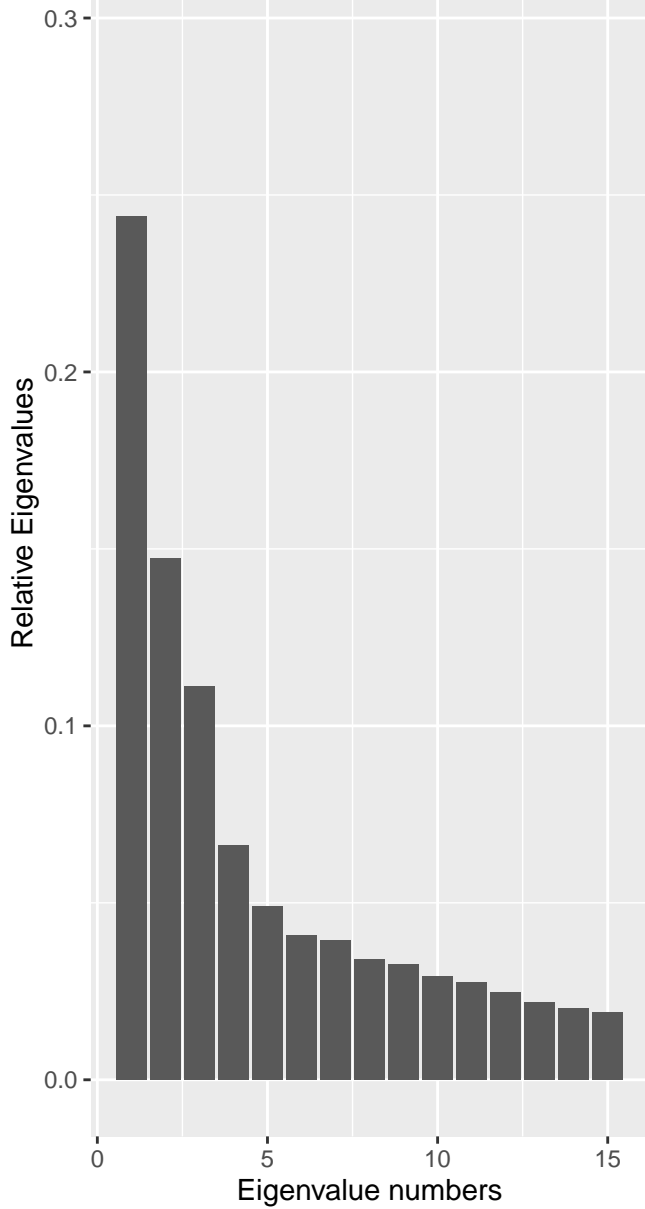

Supplement: Additional file 13: Figure S8. — Relative variances explained by the PCoA axes from the PCoA analyses A) Relative variances explained by the PCoA axes used in the Additional file 7: Figure S7ABC. B) Relative variances explained by the PCoA axes used in the adjusted sample plot in Additional file 7: Figure S7D, which had its Bacteroides and Prevotella relative abundances removed. (PDF 4 kb) [file 40168_2016_160_MOESM13_ESM.pdf]
