# Supplementary material for: Interpreting Prevotella and Bacteroides as biomarkers of diet and lifestyle
Source: Microbiome. 2016 Apr 12;4:15. doi: 10.1186/s40168-016-0160-7 (PMC4828855; doi:10.1186/s40168-016-0160-7)
Supplement: Additional file 4: Table S1. — Summary of the data received for each data set, as well as the format in which the data was used for the multivariate and proportionality analyses. (DOCX 81 kb) [file 40168_2016_160_MOESM4_ESM.docx]

| Study | Data Received | Our Processing | Used in SparCC | Used in Spearman | Used in DESeq2 |
| --- | --- | --- | --- | --- | --- |
| Ou – African Americans vs. Native Africans | Relative abundances | - | Multiplied relative abundances by 10,000 | Used original relative abundance | N/A |
| Arumugam –Mixed Europe and Asia | Relative abundances | - | Multiplied relative abundances by 10,000 | Used original relative abundance | N/A |
| Arumugam - Europe | Relative abundances | - | Multiplied relative abundances by 10,000 | Used original relative abundance | N/A |
| Human Microbiome Project - United States | Genus counts | - | Used original counts | Used relative abundance | Used original count data |
| Tyakht – Russia | Genus counts scaled by read length | Scaled to Relative Abundance | Multiplied relative abundances by 10,000 | Used relative abundance | N/A |
| Yatsunenko – Malawi, Venezuela, United States | Sequence counts | Identified sequences through GAST, Removed children <13 | Used original counts | Used relative abundance | Used original count data |

N/A the test was not used on this data set.
