# Supplementary material for: Interpreting Prevotella and Bacteroides as biomarkers of diet and lifestyle
Source: Microbiome. 2016 Apr 12;4:15. doi: 10.1186/s40168-016-0160-7 (PMC4828855; doi:10.1186/s40168-016-0160-7)
Supplement: Additional file 5: — RDATAand RMD.tar.gz is a zipped file of all the data and R markdown files to reproduce all the analyses done in the article and is the files can be found at : <http://purl.stanford.edu/fs506ff9976>. (ZIP 11504 kb) [file 40168_2016_160_MOESM5_ESM.zip › RDATAandRMD.tar.gz!/40168_2016_160_MOESM13_ESM/Biomarker_Proportions.html]

Biomarker\_Proportions


# Biomarker\_Proportions

#### *Anastassia Gorvitovskaia and Susan Holmes*

#### *December 31, 2015*

```
library(phyloseq)
library(ggplot2)
###This command needs to be changed to reflect the directory where
###the biomarkers_physeq.RData has been downloaded
load("/Users/susan/Dropbox/CaseStudies/biomarkers_physeq.RData")
##The compositions package is required for this analysis
#install.packages("compositions")
library("compositions")
```

```
## Loading required package: tensorA
```

```
## 
## Attaching package: 'tensorA'
```

```
## The following object is masked from 'package:base':
## 
##     norm
```

```
## Loading required package: robustbase
```

```
## Loading required package: energy
```

```
## Loading required package: bayesm
```

```
## Welcome to compositions, a package for compositional data analysis.
## Find an intro with "? compositions"
```

```
## 
## Attaching package: 'compositions'
```

```
## The following objects are masked from 'package:stats':
## 
##     cor, cov, dist, var
```

```
## The following objects are masked from 'package:base':
## 
##     %*%, scale, scale.default
```

```
###Extract the OTU data from the phyloseq object
otu3=otu_table(physeq3)
dim(otu3)
```

```
## [1] 484 747
```

```
Rel=t(otu3) 
nearzeros=which(Rel<(10^(-7)),arr.ind=TRUE)
nz=nrow(nearzeros)
set.seed(2015311211)
replacenz=runif(nz,min=10^(-9),max=2*10^(-9))
Rel[nearzeros]=replacenz
###Check on the values replacing the very small cells
summary(replacenz)
```

```
##      Min.   1st Qu.    Median      Mean   3rd Qu.      Max. 
## 1.000e-09 1.250e-09 1.501e-09 1.500e-09 1.750e-09 2.000e-09
```

```
# The variance of the log-ratios (i.e., the variation array)
Rel.vlr=variation(acomp(Rel)) 
Rel.clr     <- as.data.frame(clr(Rel)) 
# The variance of each variable
Rel.clr.var <- apply(Rel.clr, 2, var) 
Rel.phi     <- sweep(Rel.vlr, 2, Rel.clr.var, FUN="/")
# We want it to be taxa by taxa (484 by 484)
dim(Rel.phi)
```

```
## [1] 484 484
```

```
below0.01=which(Rel.phi<0.01,arr.ind=TRUE)
sig0.01=below0.01[below0.01[,1]!=below0.01[,2],]
dim(sig0.01)
```

```
## [1] 26  2
```

```
table(sig0.01[,2])
```

```
## 
##  98 102 112 198 224 284 319 390 
##   2   4   4   1   4   4   1   6
```

```
sigTaxa=unique(as.vector(sig0.01))
tax_table(physeq3)[sigTaxa,2:6]
```

```
## Taxonomy Table:     [8 taxa by 5 taxonomic ranks]:
##        Phylum                          Class                
## otu319 "Proteobacteria"                "Alphaproteobacteria"
## otu390 "Proteobacteria"                "Deltaproteobacteria"
## otu98  "Chlorobi"                      "Chlorobia"          
## otu112 "Firmicutes"                    "Bacilli"            
## otu284 "Firmicutes"                    "Clostridia"         
## otu102 "Cyanobacteria_Family_II_GpIIa" NA                   
## otu224 "Firmicutes"                    "Clostridia"         
## otu198 "Firmicutes"                    "Clostridia"         
##        Order                    Family                   
## otu319 "Rhizobiales"            "Rhizobiaceae"           
## otu390 "Desulfuromonadales"     "Geobacteraceae"         
## otu98  "Chlorobiales"           "Chlorobiaceae"          
## otu112 "Bacillales"             "Listeriaceae"           
## otu284 "Thermoanaerobacterales" "Thermoanaerobacteraceae"
## otu102 NA                       NA                       
## otu224 "Clostridiales"          "Peptococcaceae"         
## otu198 "Clostridiales"          "Incertae_Sedis_XVIII"   
##        Genus               
## otu319 "Rhizobium"         
## otu390 "Geobacter"         
## otu98  "Chlorobium"        
## otu112 "Listeria"          
## otu284 "Thermoanaerobacter"
## otu102 NA                  
## otu224 "Desulfotomaculum"  
## otu198 "Symbiobacterium"
```

## Amount of samples with values for the significant taxa greater than 0

```
for (j in 1:length(sigTaxa)) {
  val=length(otu_table(physeq3)[sigTaxa[j],otu_table(physeq3)[sigTaxa[j],]>0])
  cat("Row ", sigTaxa[j], " : ", val, " : ", val/747,"\n")
}
```

```
## Row  319  :  85  :  0.1137885 
## Row  390  :  85  :  0.1137885 
## Row  98  :  85  :  0.1137885 
## Row  112  :  85  :  0.1137885 
## Row  284  :  85  :  0.1137885 
## Row  102  :  85  :  0.1137885 
## Row  224  :  85  :  0.1137885 
## Row  198  :  85  :  0.1137885
```

## Mean, Minimum and Maximum Values for the significant taxa

```
for (j in 1:length(sigTaxa)) {
  mean_forTaxa=mean(otu_table(physeq3)[sigTaxa[j],])
  min_forTaxa=min(otu_table(physeq3)[sigTaxa[j],])
  max_forTaxa=max(otu_table(physeq3)[sigTaxa[j],])
  cat("Row ", sigTaxa[j], " Mean : ", mean_forTaxa, " Min : ", min_forTaxa, " Max : ", max_forTaxa,"\n")
}
```

```
## Row  319  Mean :  2.304432e-06  Min :  0  Max :  0.0001074907 
## Row  390  Mean :  3.673257e-06  Min :  0  Max :  0.0002007957 
## Row  98  Mean :  2.771477e-06  Min :  0  Max :  0.0001613967 
## Row  112  Mean :  4.203196e-06  Min :  0  Max :  0.0001958568 
## Row  284  Mean :  3.925827e-06  Min :  0  Max :  0.0001587737 
## Row  102  Mean :  3.204389e-06  Min :  0  Max :  0.0001882159 
## Row  224  Mean :  3.809479e-06  Min :  0  Max :  0.0001316869 
## Row  198  Mean :  3.064652e-06  Min :  0  Max :  0.0001240854
```
