# Supplementary material for: Interpreting Prevotella and Bacteroides as biomarkers of diet and lifestyle
Source: Microbiome. 2016 Apr 12;4:15. doi: 10.1186/s40168-016-0160-7 (PMC4828855; doi:10.1186/s40168-016-0160-7)
Supplement: Additional file 5: — RDATAand RMD.tar.gz is a zipped file of all the data and R markdown files to reproduce all the analyses done in the article and is the files can be found at : <http://purl.stanford.edu/fs506ff9976>. (ZIP 11504 kb) [file 40168_2016_160_MOESM5_ESM.zip › RDATAandRMD.tar.gz!/40168_2016_160_MOESM13_ESM/Biomarkers_Figures_withellipses_2_12_16.html]

Biomarker\_Figures


# Biomarker\_Figures

#### *Susan Holmes*

#### *February 12, 2015*

```
library(phyloseq)
```

```
## Warning: replacing previous import by 'ggplot2::Position' when loading
## 'phyloseq'
```

```
## Warning: replacing previous import by 'scales::alpha' when loading 'phyloseq'
```

```
library(ggplot2)
library(dplyr)
```

```
## 
## Attaching package: 'dplyr'
```

```
## The following objects are masked from 'package:stats':
## 
##     filter, lag
```

```
## The following objects are masked from 'package:base':
## 
##     intersect, setdiff, setequal, union
```

```
library(reshape2)
library(ellipse)
library(RColorBrewer)
library(gridExtra)
setwd("/Users/susan/Dropbox/EnterotypeSH/DataFilesforUpload")
load("/Users/susan/Dropbox/CaseStudies/biomarkers_physeq.RData")
colors <- c("black", "red", "blue","light blue", "light green", "orange", "deeppink1")
pchs <- c(20,5)
dimnames(sample_data(physeq3))[[2]]=c("Location","data",      "Origin","Dominance","PB_Ratio","bact_abun","prev_abun")
abundances=data.frame(xax=seq(0,1,length=747),sample_data(physeq3)[,c(2,5,6,7)])
names(abundances)[2]="Data_Origin"
# Bacteroides "otu44"
# Prevotella "otu62"
bact_otu <- 44
prev_otu <- 62
```

# Figure 2 with P/B ratio calculation

## Creating a Bacteroides, Prevotella, P/B Distribution

```
#Figure 2 Prevotella ratio distributions 
xaxis=rep(c((1:41)/41,(1:85)/85,(1:210)/210,(1:24)/24,(1:96)/96,(1:291)/291),3)
datalong=melt(abundances,id.vars="Data_Origin")
datasort=datalong %>%
   arrange(variable,Data_Origin,desc(value))
datas=datasort[datasort$variable !="xax",]
datas=cbind(datas,xaxis)
colors <- c("black", "red", "blue","light blue", "light green", "orange", "deeppink1")
#arum41 arum85    hmp     ou   russ   yats
```

```
###pp1 makes the main Figure 2
pp1=ggplot(subset(datas,variable=="PB_Ratio"),aes(x=xaxis,y=value,colour=Data_Origin))+geom_point()+ geom_line(size=1.2)+
  geom_line(aes(x=sort(xaxis),                y=sort(value,decreasing=TRUE),color="all"),size=1.5)+
  scale_color_manual(values=colors)
pp1=pp1+scale_y_continuous("Prevotella/(Prevotella + Bacteroides)")+
  scale_x_continuous("Proportion of Samples")
#========================================================
#pdf("FinalFigures/MainFigure2.pdf",width=7,height=5.5)
pp1
```

```
#dev.off()
#========================================================
```

## Bacteroides relative abundances by data origin

Supplemental Figure 2.

```
pp2=ggplot(subset(datas,variable=="bact_abun"),aes(x=xaxis,y=value,colour=Data_Origin))+geom_point()+ geom_line(size=1.2)+
  geom_line(aes(x=sort(xaxis),                y=sort(value,decreasing=TRUE),color="all"),size=1.5)+
  scale_color_manual(values=colors)
pp2=pp2+scale_y_continuous("Bacteroides")+
  scale_x_continuous("Proportion of Samples")
#######################
#pdf("FinalFigures/SuppFigure2.pdf",width=7,height=5.5)
pp2
```

```
#dev.off()
########################
```

## Prevotella relative abundances by data origin, Supplemental Figure 3.

```
pp3=ggplot(subset(datas,variable=="prev_abun"),aes(x=xaxis,y=value,colour=Data_Origin))+geom_point()+ geom_line(size=1.2)+
  geom_line(aes(x=sort(xaxis),                y=sort(value,decreasing=TRUE),color="all"),size=1.5) +
  scale_color_manual(values=c(colors))
pp3=pp3+scale_y_continuous("Prevotella")+
  scale_x_continuous("Proportion of Samples")
##======================================================
#pdf("FinalFigures/SuppFigure3.pdf",width=7,height=5.5)
pp3
```

```
#dev.off()
##======================================================
```

# MDS Analysis of All the Data: Supplementary Figure 1

```
Ent.ord.mds <- ordinate(physeq3, "MDS", "bray")
p3o = plot_ordination(physeq3, Ent.ord.mds, type = "samples", color = "Origin", shape="Origin")
p3o=p3o +  geom_point(aes(shape=Origin),size=3,fill="white",alpha=0.5) +
        scale_shape_manual(values=c(9:10,12,15:18,21:25))
  
  #scale_shape_manual(values=1:12)
#########################################
###Important: scale respects proportion of variability
#######################################
#pdf("FinalFigures/SuppFigure1_MDS_All.pdf",width=7.2,height=5)
p3o
```

```
#dev.off()
############################################
```

As the data show clusters the different data sets separately according to the different studies and the two US studies cluster separately, we are removing the Yatsunenko data.

# MDS Analysis without the Yatsunenko Data (Malawi, Venezuela, US)

Figure 1 PCoA plots using the Bray distance metric with all the samples except for the Yatsunenko study.  
A) Samples colored by their most prominent taxon. If the sample is dominated neither by Prevotella nor Bacteroides, it is classified as other with an ellipse for each subgroup.

2. Samples are colored by their value for the Prevotella ratio (relative abundance of Prevotella / [Bacteroides + Prevotella]) on a spectrum with red indicating no Prevotella and purple no Bacteroides.
3. Samples are colored by population of origin.
4. The Bray distance has been recalculated without the rel. abundances of Bacteroides and Prevotella. Samples are colored by most prominent taxon in the original samples distributions (same as in plot A) and ellipses shown in the same way.

## Making the core 4 images of Main Figure 1

```
#####Plot (A) without ellipses
noYats_sample_names <- rownames(sample_data(physeq3)[sample_data(physeq3)[,2]!="yats",])
noYats_physeq=prune_samples(noYats_sample_names,physeq3)
noYats_Ent.ord.mds <- ordinate(noYats_physeq, "MDS", "bray")
###Dominance Plots
p_noY_d = plot_ordination(noYats_physeq, noYats_Ent.ord.mds, type = "samples", color = "Dominance", shape="Dominance")
p_noY_d =p_noY_d + geom_point(aes(shape=Dominance),size=3,alpha=0.5)+
  scale_colour_brewer(palette ="Set2",name="Dominance",
        labels=c("Bacteroides", "Other", "Prevotella"))
p_noY_d
```

```
#Plot 1A with ellipses
#group variable
groupvar=sample_data(noYats_physeq)$Dominance
#continuous variable
pbr=sample_data(noYats_physeq)$PB_Ratio
continvar=pbr
x=noYats_Ent.ord.mds$vectors[,1]
y=noYats_Ent.ord.mds$vectors[,2]
percentinertia=round(noYats_Ent.ord.mds$values[1:3,2]*100)
colorpal=brewer.pal(4, "Spectral")
#pellipse1A=plotellipseord(x,y,pbr,dominance,eigs)
df <- data.frame(x=x, y=y,group=factor(groupvar),continvar)
df_ell <- data.frame() 
for(g in levels(df$group)){df_ell <- rbind(df_ell, cbind(as.data.frame(with(df[df$group==g,], ellipse(cor(x, y),scale=c(sd(x),sd(y)),centre=c(mean(x),mean(y))))),group=g))} 
pellipse1A <- ggplot(data=df, aes(x=x, y=y,colour=group,shape=group)) + 
  geom_point() + 
  geom_path(data=df_ell, aes(x=x, y=y,colour=group)) +                
  xlab(paste("Axis.1 [",percentinertia[1],"%]",sep="")) +
  ylab(paste("Axis.2 [",15.6,"%]",sep="")) +
  scale_colour_brewer(palette ="Set2",name="Dominance",
        labels=c("Bacteroides", "Other", "Prevotella"))+
  scale_shape_manual(values=c(16, 17, 15),
                     name="Dominance",
        labels=c("Bacteroides", "Other", "Prevotella")) +
   annotate("text",label="(A)",x = -0.65, y =0.405 , size=7)
#scale_colour_manual(values=colorpal) 
###Colors BrBG, PiYG, PRGn, PuOr, RdBu, RdGy, RdYlBu, RdYlGn
print(pellipse1A)
```

## Using the Prevotella/(B+P) ratio as a gradient.

```
p_noY_pb = plot_ordination(noYats_physeq, noYats_Ent.ord.mds, type = "samples", color = "PB_Ratio")
p_noY_pb=p_noY_pb + scale_colour_gradientn(colours = rainbow(7)[c(1,3:7)])+
   annotate("text",label="(B)",x = -0.5, y =0.29 , size=7)
p_noY_pb
```

```
p_noY_o = plot_ordination(noYats_physeq, noYats_Ent.ord.mds, type = "samples", color = "Origin", shape="Origin")
p_noY_o=p_noY_o +  geom_point(aes(shape=Origin),size=3,fill="white",alpha=0.5) +
        scale_shape_manual(values=c(15:18,21:25))
#guide = guide_legend(key.width = 0.1))
#+ theme(legend.key.width=unit(0.2,"cm"))
p_noY_o=p_noY_o +
   annotate("text",label="(C)",x = -0.5, y =0.3 , size=7)
p_noY_o
```

## Analysis without the Bacteroides nor Prevotella.

```
noBP_otus=paste("otu",(1:484)[-c(prev_otu,bact_otu)],sep="")
noBP_physeq=prune_taxa(noBP_otus,noYats_physeq)
noBP.mds=ordinate(noBP_physeq, "MDS", "bray")
p_noY_noBP_d = plot_ordination(noBP_physeq, noBP.mds, type = "samples", color = "Dominance", shape="Dominance",axes=c(1,2))
p_noY_noBP_d +  geom_point(aes(shape=Dominance),size=3,alpha=0.5)
```

```
  #scale_shape_manual(values=1:3)
```

```
groupvar=sample_data(noBP_physeq)$Dominance
#continuous variable
pbr=sample_data(noBP_physeq)$PB_Ratio
continvar=pbr
x=noBP.mds$vectors[,1]
y=noBP.mds$vectors[,2]
percentinertia=round(noBP.mds$values[1:3,2]*100)
colorpal=brewer.pal(4, "Spectral")
#pellipse1B=plotellipseord(x,y,pbr,dominance,eigs)
df <- data.frame(x=x, y=y,group=factor(groupvar),continvar)
df_ell <- data.frame() 
for   (g in levels(df$group)){
        df_ell <- rbind(df_ell,      cbind(as.data.frame(with(df[df$group==g,],                   ellipse(cor(x, y),scale=c(sd(x),sd(y)),                             centre=c(mean(x),mean(y))))),group=g))
} 
#####################################################
##The ellipse functions plots a 95% ellipse
##if the data were bivariate normal with the
## given standard deviations, correlation and means
#####################################################
pellipse1D <- ggplot(data=df, aes(x=x, y=y,colour=group,shape=group)) + 
  geom_point() + 
  geom_path(data=df_ell, aes(x=x, y=y,colour=group)) +                             #scale_colour_manual(values=colorpal) +
  xlab(paste("Axis 1: [",23.6,"%]",sep="")) +
  ylab(paste("Axis 2: [",percentinertia[2],"%]",sep="")) +
  scale_colour_brewer(palette ="Set2",name="Dominance",
        labels=c("Bacteroides", "Other", "Prevotella")) +
  scale_shape_manual(values=c(16, 17, 15),name="Dominance",
        labels=c("Bacteroides", "Other", "Prevotella")) 
pellipse1D=pellipse1D+ annotate("text",label="(D)",x = -0.65, y =0.38 , size=7)
print(pellipse1D)
```

Four figures in one page for pdf file.

```
####### (A):By dominant taxa
#A1=p_noY_d + scale_shape_manual(values=1:3)
#######  (B): PSB gradient
B1=p_noY_pb 
#+ scale_colour_gradientn(colours = rainbow(7)[c(1,3:7)])
###### (C):Data origin of samples
C1=p_noY_o 
#+ scale_shape_manual(values=1:9)
######  (D): Bacteroides and Prevotella abundances removed, same
######coloring as in (A)
#D1=p_noY_noBP_d + scale_shape_manual(values=1:3)
####################################
#pdf("FinalFigures/MainFigure1.pdf",width=7.5,height=15)
#grid.arrange(pellipse1A,B1,C1,pellipse1D,ncol=1,newpage=FALSE)
#dev.off()
########################################
```

# Correlation calculations with the first axis

Correlations between the first axis and bacteroides abundance and between the second axis and the prevotella abundance are substantial (and statistically significant) as can be seen by these permutation tests.

```
###Correlation study for first and second axes.
axis1=noYats_Ent.ord.mds$vectors[,1]
axis2=noYats_Ent.ord.mds$vectors[,2]
bacta=sample_data(noYats_physeq)$bact_abun
prevota=sample_data(noYats_physeq)$prev_abun
cor(axis1,bacta)
```

```
## [1] 0.93
```

```
cor(axis2,prevota)
```

```
## [1] -0.796
```

```
corfun <- function(x=axis1,y=bacta) {
    return(cor(x, sample(y)))
}
cor0=cor(axis1,bacta)
testc <- c( cor0,replicate(9999, corfun()) )
sum( testc >= testc[1] )/length(testc)
```

```
## [1] 1e-04
```

```
cor0=cor(axis2,prevota)
testc <- c( cor0,replicate(9999, corfun(x=axis2,y=prevota)) )
sum( testc <= testc[1] )/length(testc)
```

```
## [1] 1e-04
```

# NMDS Analysis without the Yatsunenko Data (Malawi, Venezuela, US)

## Making of the core 4 images for Supplemental Figure 4

```
noYats_Ent.ord.nmds <- ordinate(noYats_physeq, "NMDS", "bray")
```

```
## Run 0 stress 0.165 
## Run 1 stress 0.181 
## Run 2 stress 0.182 
## Run 3 stress 0.188 
## Run 4 stress 0.176 
## Run 5 stress 0.175 
## Run 6 stress 0.176 
## Run 7 stress 0.181 
## Run 8 stress 0.173 
## Run 9 stress 0.173 
## Run 10 stress 0.176 
## Run 11 stress 0.176 
## Run 12 stress 0.178 
## Run 13 stress 0.175 
## Run 14 stress 0.176 
## Run 15 stress 0.172 
## Run 16 stress 0.175 
## Run 17 stress 0.181 
## Run 18 stress 0.182 
## Run 19 stress 0.18 
## Run 20 stress 0.173
```

```
pn_noY_d = plot_ordination(noYats_physeq, noYats_Ent.ord.nmds, type = "samples", color = "Dominance", shape="Dominance")
As4=pn_noY_d + scale_shape_manual(values=c(16, 17, 15),name="Dominance",
        labels=c("Bacteroides", "Other", "Prevotella")) +
  scale_colour_brewer(palette ="Set2",name="Dominance",
        labels=c("Bacteroides", "Other", "Prevotella"))
 # scale_shape_manual(values=1:3)
As4=As4+  annotate("text",label="(A)",x = -1.83, y =2.2 , size=7)
pn_noY_pb = plot_ordination(noYats_physeq,noYats_Ent.ord.nmds, type = "samples", color = "PB_Ratio")
Bs4=pn_noY_pb + scale_colour_gradientn(colours = rainbow(7)[c(1,3:7)])
Bs4=Bs4+  annotate("text",label="(B)",x = -1.83, y =2.2 , size=7)
pn_noY_o = plot_ordination(noYats_physeq, noYats_Ent.ord.nmds, type = "samples", color = "Origin", shape="Origin")
Cs4=pn_noY_o +  geom_point(aes(shape=Origin),size=3,fill="white",alpha=0.5) +
        scale_shape_manual(values=c(15:18,21:25))
#scale_shape_manual(values=1:9)
Cs4=Cs4+  annotate("text",label="(C)",x = -1.83, y =2.2 , size=7)
noBP.nmds=ordinate(noBP_physeq, "NMDS", "bray")
```

```
## Run 0 stress 0.196 
## Run 1 stress 0.217 
## Run 2 stress 0.221 
## Run 3 stress 0.215 
## Run 4 stress 0.215 
## Run 5 stress 0.222 
## Run 6 stress 0.215 
## Run 7 stress 0.198 
## Run 8 stress 0.222 
## Run 9 stress 0.219 
## Run 10 stress 0.212 
## Run 11 stress 0.219 
## Run 12 stress 0.214 
## Run 13 stress 0.217 
## Run 14 stress 0.211 
## Run 15 stress 0.217 
## Run 16 stress 0.211 
## Run 17 stress 0.208 
## Run 18 stress 0.217 
## Run 19 stress 0.22 
## Run 20 stress 0.221
```

```
pn_noY_noBP_d = plot_ordination(noBP_physeq, noBP.nmds, type = "samples", color = "Dominance", shape="Dominance",axes=c(1,2))
Ds4=pn_noY_noBP_d +  scale_shape_manual(values=c(16, 17, 15),name="Dominance",
        labels=c("Bacteroides", "Other", "Prevotella")) +
  scale_colour_brewer(palette ="Set2",name="Dominance",
        labels=c("Bacteroides", "Other", "Prevotella"))
  #scale_shape_manual(values=1:3)
Ds4=Ds4+  annotate("text",label="(D)",x = -3.3, y =2.2 , size=7)
As4
```

```
Bs4
```

```
Cs4
```

```
Ds4
```

```
#pdf("FinalFigures/SuppFigure4_NMDS_noY.pdf",width=14,height=11)
#grid.arrange(As4,Bs4,Cs4,Ds4,ncol=2,newpage=FALSE)
#dev.off()
```

# Looking at the 3rd and 4th axis without the Yatsunenko data

## MDS Analysis without the Yatsunenko Data (Malawi, Venezuela, US)

```
p_noY_d34 = plot_ordination(noYats_physeq, noYats_Ent.ord.mds, type = "samples", color = "Dominance", shape="Dominance", axes=c(3,4))
As5= p_noY_d34 + scale_shape_manual(values=c(16, 17, 15),name="Dominance",
        labels=c("Bacteroides", "Other", "Prevotella")) +
  scale_colour_brewer(palette ="Set2",name="Dominance",
        labels=c("Bacteroides", "Other", "Prevotella"))+
 annotate("text",label="(A)",x = -0.35, y =0.2 , size=7)
# scale_shape_manual(values=1:3)

p_noY_pb34 = plot_ordination(noYats_physeq, noYats_Ent.ord.mds, type = "samples", color = "PB_Ratio", axes=c(3,4))
Bs5= p_noY_pb34 + scale_colour_gradientn(colours = rainbow(7)[c(1,3:7)])+annotate("text",label="(B)",x = -0.35, y =0.2 , size=7)

p_noY_o34 = plot_ordination(noYats_physeq, noYats_Ent.ord.mds, type = "samples", color = "Origin", shape="Origin", axes=c(3,4))
Cs5=p_noY_o34 +  geom_point(aes(shape=Origin),size=3,fill="white",alpha=0.5) +
         scale_shape_manual(values=c(15:18,21:25))+
annotate("text",label="(C)",x = -0.3, y =0.2 , size=7)
#  scale_shape_manual(values=1:9)
p_noY_noBP_d34 = plot_ordination(noBP_physeq, noBP.mds, type = "samples", color = "Dominance", shape="Dominance", axes=c(3,4))
Ds5= p_noY_noBP_d34 +  scale_shape_manual(values=c(16, 17, 15),name="Dominance",
        labels=c("Bacteroides", "Other", "Prevotella")) +
  scale_colour_brewer(palette ="Set2",name="Dominance",
        labels=c("Bacteroides", "Other", "Prevotella"))+
annotate("text",label="(D)",x = -0.3, y =0.43 , size=7)
As5
```

```
Bs5
```

```
Cs5
```

```
Ds5
```

```
#########################################################
#pdf("FinalFigures/SuppFigure5_PCoA_34.pdf",width=13,height=11)
#grid.arrange(As5,Bs5,Cs5,Ds5,ncol=2,newpage=FALSE)
#dev.off()
```

```
dfev=data.frame(Eigenvalues=noYats_Ent.ord.mds$values[1:15,2],number=1:15)
bp1=ggplot(dfev,aes(x=number,y=Eigenvalues))+geom_bar(stat="identity")+ ylim(0,0.38)+ylab("Relative Eigenvalues")+
annotate("text",label="(A)",x = 8, y =0.35 , size=7)+
  xlab("Eigenvalue numbers")

dfev2=data.frame(Eigenvalues=noBP.mds$values[1:15,2],number=1:15)
bp2=ggplot(dfev2,aes(x=number,y=Eigenvalues))+geom_bar(stat="identity")+ ylim(0,0.38)+ylab("Relative Eigenvalues")+
  xlab("Eigenvalue numbers")+
annotate("text",label="(B)",x = 8, y =0.35 , size=7)
#pdf("FinalFigures/SuppFigure6_Eigenvalue_Barplots.pdf")
grid.arrange(bp1,bp2,ncol=2,newpage=FALSE)
```

```
#dev.off()
```

# Analysis of Yatsunenko data alone

## MDS Analysis of the Yatsunenko Data (Malawi, Venezuela, US)

## Making of the core 4 images

```
yats_sample_names <- rownames(sample_data(physeq3)[sample_data(physeq3)[,2]=="yats",])
yats_physeq=prune_samples(yats_sample_names,physeq3)
yats_Ent.ord.mds <- ordinate(yats_physeq, "MDS", "bray")

p_y_d = plot_ordination(yats_physeq, yats_Ent.ord.mds, type = "samples", color = "Dominance", shape="Dominance")
p_y_d = p_y_d +  scale_shape_manual(values=c(16, 17, 15),name="Dominance",
        labels=c("Bacteroides", "Other", "Prevotella")) +
  scale_colour_brewer(palette ="Set2",name="Dominance",
        labels=c("Bacteroides", "Other", "Prevotella"))+
annotate("text",label="(A)",x = -0.5, y =0.3 , size=7)
p_y_pb = plot_ordination(yats_physeq, yats_Ent.ord.mds, type = "samples", color = "PB_Ratio")
p_y_pb = p_y_pb + scale_colour_gradientn(colours = rainbow(7)[c(1,3:7)])+
annotate("text",label="(B)",x = -0.5, y =0.3 , size=7)

p_y_o = plot_ordination(yats_physeq, yats_Ent.ord.mds, type = "samples", color = "Origin", shape="Origin")
p_y_o = p_y_o + scale_shape_manual(values=1:9)+
annotate("text",label="(C)",x = -0.5, y =0.3 , size=7)
noBP_otus=paste("otu",(1:484)[-c(prev_otu,bact_otu)],sep="")
yats_noBP_physeq=prune_taxa(noBP_otus,yats_physeq)
yats_noBP.mds=ordinate(yats_noBP_physeq, "MDS", "bray")

p_y_noBP_d = plot_ordination(yats_noBP_physeq, yats_noBP.mds, type = "samples", color = "Dominance", shape="Dominance",axes=c(1,2))
p_y_noBP_d=p_y_noBP_d +   scale_shape_manual(values=c(16, 17, 15),name="Dominance",
        labels=c("Bacteroides", "Other", "Prevotella")) +
  scale_colour_brewer(palette ="Set2",name="Dominance",
        labels=c("Bacteroides", "Other", "Prevotella"))+
annotate("text",label="(D)",x = -0.47, y =0.22 , size=7)
p_y_d
```

```
p_y_pb
```

```
p_y_o
```

```
p_y_noBP_d
```

```
#########################################################
#pdf("FinalFigures/SuppFigure7_PCoABray_Yats.pdf",height=14,width=10)
#grid.arrange(p_y_d,p_y_pb,p_y_o,p_y_noBP_d,ncol=1,newpage=FALSE)
#dev.off()
```

```

```
dfevy=data.frame(Eigenvalues=yats_Ent.ord.mds$values[1:15,2],
                number=1:15)
bp1y=ggplot(dfevy,aes(x=number,y=Eigenvalues))+geom_bar(stat="identity")+
annotate("text",label="(A)",x = 8, y =0.35 , size=7)+
 ylim(0,0.32)+ylab("Relative Eigenvalues")+
annotate("text",label="(A)",x = 8, y =0.32 , size=7)+
  xlab("Eigenvalue numbers")
######Yats data without Bact nor Prevotella
dfevy2=data.frame(Eigenvalues=yats_noBP.mds$values[1:15,2],number=1:15)
bp2y=ggplot(dfevy2,aes(x=number,y=Eigenvalues))+geom_bar(stat="identity")+
annotate("text",label="(B)",x = 8, y =0.32 , size=7)+
ylab("Relative Eigenvalues")+
 ylim(0,0.32)+
  xlab("Eigenvalue numbers")

########################################################
#pdf("FinalFigures/SuppFigure8_Eigenvalue_Barplots_Yats.pdf")
grid.arrange(bp1y,bp2y,ncol=2,newpage=FALSE)
```

```
## Warning: Removed 1 rows containing missing values (geom_text).
```

```
#dev.off()
##################################################
```

# NMDS Analysis of the Yatsunenko Data (Malawi, Venezuela, US)

## Making of the core 4 images

```
yats_Ent.ord.nmds <- ordinate(yats_physeq, "NMDS", "bray")
```

```
## Run 0 stress 0.178 
## Run 1 stress 0.179 
## Run 2 stress 0.178 
## ... New best solution
## ... procrustes: rmse 0.0157  max resid 0.1 
## Run 3 stress 0.177 
## ... New best solution
## ... procrustes: rmse 0.0145  max resid 0.0997 
## Run 4 stress 0.215 
## Run 5 stress 0.191 
## Run 6 stress 0.185 
## Run 7 stress 0.177 
## ... New best solution
## ... procrustes: rmse 0.00511  max resid 0.0599 
## Run 8 stress 0.186 
## Run 9 stress 0.18 
## Run 10 stress 0.179 
## Run 11 stress 0.185 
## Run 12 stress 0.185 
## Run 13 stress 0.177 
## ... procrustes: rmse 0.0109  max resid 0.0991 
## Run 14 stress 0.418 
## Run 15 stress 0.179 
## Run 16 stress 0.178 
## Run 17 stress 0.18 
## Run 18 stress 0.178 
## Run 19 stress 0.18 
## Run 20 stress 0.185
```

```
pn_y_d = plot_ordination(yats_physeq, yats_Ent.ord.nmds, type = "samples", color = "Dominance", shape="Dominance")
pn_y_d = pn_y_d +    scale_shape_manual(values=c(16, 17, 15),name="Dominance",
        labels=c("Bacteroides", "Other", "Prevotella")) +
  scale_colour_brewer(palette ="Set2",name="Dominance",
        labels=c("Bacteroides", "Other", "Prevotella"))+
  annotate("text",label="(A)",x = -1.3, y =0.77 , size=7)

pn_y_pb = plot_ordination(yats_physeq,yats_Ent.ord.nmds, type = "samples", color = "PB_Ratio")
pn_y_pb = pn_y_pb + scale_colour_gradientn(colours = rainbow(7)[c(1,3:7)])+
annotate("text",label="(B)",x = -1.3, y =0.77 , size=7)

pn_y_o = plot_ordination(yats_physeq, yats_Ent.ord.nmds, type = "samples", color = "Origin", shape="Origin")
pn_y_o = pn_y_o + scale_shape_manual(values=1:9)+
annotate("text",label="(C)",x = -1.3, y =0.77 , size=7)

yats_noBP.nmds=ordinate(noBP_physeq, "NMDS", "bray")
```

```
## Run 0 stress 0.196 
## Run 1 stress 0.215 
## Run 2 stress 0.219 
## Run 3 stress 0.221 
## Run 4 stress 0.222 
## Run 5 stress 0.214 
## Run 6 stress 0.223 
## Run 7 stress 0.229 
## Run 8 stress 0.225 
## Run 9 stress 0.215 
## Run 10 stress 0.224 
## Run 11 stress 0.21 
## Run 12 stress 0.419 
## Run 13 stress 0.221 
## Run 14 stress 0.215 
## Run 15 stress 0.22 
## Run 16 stress 0.224 
## Run 17 stress 0.218 
## Run 18 stress 0.205 
## Run 19 stress 0.212 
## Run 20 stress 0.216
```

```
####Colored and shaped by dominance
pn_y_noBP_d = plot_ordination(noBP_physeq, yats_noBP.nmds, type = "samples", color = "Dominance", shape="Dominance",axes=c(1,2))
pn_y_noBP_d = pn_y_noBP_d +    scale_shape_manual(values=c(16, 17, 15),name="Dominance",
        labels=c("Bacteroides", "Other", "Prevotella")) +
  scale_colour_brewer(palette ="Set2",name="Dominance",
        labels=c("Bacteroides", "Other", "Prevotella"))+
annotate("text",label="(D)",x = -3.2, y =1.1 , size=7)
pn_y_d
```

```
pn_y_pb
```

```
pn_y_o
```

```
pn_y_noBP_d
```

```
####################################################### 
#pdf("FinalFigures/SuppFigure9_NMDS_Bray_Yats.pdf",height=15,width=10)
#grid.arrange(pn_y_d,pn_y_pb,pn_y_o,pn_y_noBP_d,ncol=1,newpage=FALSE)
#dev.off()
```

# Different Distance Methods PCoAs

```
noYats_jsd.ord.mds <- ordinate(noYats_physeq, "MDS", "jsd")
noYats_horn.ord.mds <- ordinate(noYats_physeq, "MDS", "horn")
# Basic Dominance Plots
p_noY_d_jsd = plot_ordination(noYats_physeq, noYats_jsd.ord.mds, type = "samples", color = "Dominance", shape="Dominance")
p_noY_d_jsd =p_noY_d_jsd + scale_shape_manual(values=c(16, 17, 15),name="Dominance",
        labels=c("Bacteroides", "Other", "Prevotella")) +
  scale_colour_brewer(palette ="Set2",name="Dominance",
        labels=c("Bacteroides", "Other", "Prevotella"))+
annotate("text",label="(A)",x = -0.37, y =0.21 , size=7)


p_noY_d_horn = plot_ordination(noYats_physeq, noYats_horn.ord.mds, type = "samples", color = "Dominance", shape="Dominance")
p_noY_d_horn =p_noY_d_horn + scale_shape_manual(values=c(16, 17, 15),name="Dominance",
        labels=c("Bacteroides", "Other", "Prevotella")) +
  scale_colour_brewer(palette ="Set2",name="Dominance",
        labels=c("Bacteroides", "Other", "Prevotella"))+
annotate("text",label="(B)",x = -0.66, y =0.46 , size=7)
#######################################
#pdf("FinalFigures/SuppFigure10_otherDist_MDS.pdf",width=10,height=5)
grid.arrange(p_noY_d_jsd,p_noY_d_horn,ncol=2,newpage=FALSE)
```

```
#dev.off()
```
